# Predicting the status of 35 sustainable development goal indicators in Indian villages: a semi-supervised machine learning approach for precision public policy

**DOI:** 10.1016/j.lansea.2026.100852

**Published:** 2026-09-08

**Authors:** Soohyeon Ko, Avleen S. Bijral, Abhimanyu Singh, Jeffrey C. Blossom, Sunil Rajpal, William Joe, S.V. Subramanian, Rockli Kim

**Affiliations:** a Department of Social and Behavioral Sciences, Harvard T. H. Chan School of Public Health, Boston, MA, USA; b Harvard Center for Population and Development Studies, Cambridge, MA, USA; c Microsoft AI for Good Research Lab, Redmond, WA, USA; d Dhirubhai Ambani International School, Mumbai, India; e Center for Geographic Analysis, Harvard University, Cambridge, MA, USA; f Department of Economics, FLAME University, Pune, India; g Centre for Research in Wellbeing and Happiness, FLAME University, Pune, India; h Institute of Economic Growth (IEG), Delhi, India; i Division of Health Policy and Management, College of Health Sciences, Korea University, Seoul, Republic of Korea; j Interdisciplinary Program in Precision Public Health, Department of Public Health Sciences, Graduate School of Korea University, Seoul, Republic of Korea

**Keywords:** Sustainable development goals, Social determinants of health, Villages, Multilevel modeling, Semi-supervised machine learning, Precision public policy, India

## Abstract

**Background:**

National and district-level monitoring of the Sustainable Development Goals (SDGs) in India obscures substantial inequalities at finer geographic resolutions, such as villages. While villages are central to service delivery and local governance, village-level estimates remain limited, hindering precision targeting and accountability. We estimated village-level prevalence for 35 SDG indicators in 2021, assessed their status relative to SDG targets, and identified Required Rates of Improvement (RRI) to achieve targets by 2030.

**Methods:**

We used cross-sectional data from the National Family Health Survey 2019–21 and the 2011 Census. We estimated cluster-level indicator values using multilevel modeling and linked them to census villages through a semi-supervised learning framework to generate predictions for 597,603 villages.

**Findings:**

Substantial village-level heterogeneity was observed across all SDG indicators. As of 2021, none of the villages had achieved several SDG targets, including health insurance coverage, access to basic services, women's bank account ownership, and internet use. In contrast, 99.9% of villages had achieved the target for adolescent pregnancy among girls aged 10–14 years. The largest mean RRI values were observed for access to basic services (7.61 percentage points/year), health insurance coverage among women (6.74), health insurance coverage among men (6.39), and clean fuel for cooking (6.05).

**Interpretation:**

Village-level inequalities are substantial enough to make district-level averages insufficient for SDG monitoring. Routine village-level monitoring can support precise targeting of public policies and strengthen local accountability. Sustaining gains among villages that have achieved SDG targets and accelerating improvement among those below target levels will be essential.

**Funding:**

This research was funded by 10.13039/100000865Bill & Melinda Gates Foundation (INV-002992).

**Research in context**

**Evidence before this study**

We searched PubMed from January 1, 2015, to January 17, 2026 to identify empirical studies that conducted quantitative analyses of SDG-related indicators in India. We used structured combinations of keywords including “Sustainable Development Goals”, “SDGs”, or “SDG indicators”, together with terms related to monitoring or evaluation (such as “progress”, “monitoring”, “evaluation”, or “surveillance”) and subnational geography (such as “state”, “district”, “small area”, “cluster”, “village”, or “local level”). The search yielded 113 studies. After screening titles and abstracts to assess whether analyses used quantitative empirical data, examined SDG indicators or closely related outcomes, and provided subnational estimates within India, we identified 11 papers that were relevant to the scope of our study. Of these, seven studies examined SDG-linked outcomes and reported results at subnational levels, but were limited to single domains, such as universal health coverage, reproductive and child health, violence against women, or child marriage. Two additional studies conducted quantitative assessments of progress toward SDG targets, but primarily at the state or district level. Two studies assessed SDG-related outcomes at very fine geographic scales, including villages or sub-district units; however, these analyses were confined to specific regions or states, relied on a narrow set of indicators, or were conducted as local case studies, limiting their generalizability for national SDG monitoring. We did not identify any study that produced nationally comprehensive, village-level estimates across a broad set of SDG indicators in India. The identified studies were heterogeneous in scope and methodology and were not formally assessed for risk of bias because the objective was to identify the extent and geographic scale of SDG monitoring efforts rather than to synthesize effect estimates.

**Added value of this study**

This study provides a comprehensive set of village-level estimates for a broader set of population health and social determinant–related SDG indicators across nearly 600,000 rural villages in India. By integrating cluster-level estimates from the individual-level survey data with census-based village characteristics through a semi-supervised learning framework, we overcome key data and methodological barriers that have previously precluded village-level SDG assessment. In addition to estimating 2021 indicator levels, we assess progress toward the 2030 SDG targets by identifying villages that have already met national benchmarks and by quantifying the annual Required Rates of Improvement for remaining villages to reach the targets. This analysis reveals substantial within-district, between-village heterogeneity and variation in the acceleration required to meet SDG targets that are obscured in district- or state-level assessments. By shifting the analytic focus from districts to villages, our study extends prior subnational SDG monitoring efforts and provides a more precise and policy-relevant understanding of where progress has stalled and where acceleration is most urgently needed. To facilitate translation of these findings, we also developed an online interactive dashboard (available at https://india-sdg-village-dashboard.streamlit.app/) to support evidence-informed decision-making by policymakers and other stakeholders.

**Implications of all the available evidence**

Taken together with existing national- and district-level studies, our findings indicate that aggregate SDG monitoring can obscure both the scale and the uneven distribution of unmet need at local levels in India. The substantial within-district, between-village variation and large differences in the acceleration required across villages suggest that uniform, district-wide strategies are unlikely to be sufficient for achieving the SDGs by 2030. Village-level SDG estimates can support more precise targeting of interventions, strengthen local accountability, and better align data with decentralized planning and implementation structures, such as those operating through local governance and service delivery systems. Given India's central role in global SDG attainment, incorporating village-level evidence into SDG monitoring and policy design is essential not only for national progress, but also for advancing the global commitment to leaving no one behind. Future research should incorporate updated census data, repeated measurements, and formal uncertainty quantification to further strengthen village-level SDG monitoring.

## Introduction

The Sustainable Development Goals (SDGs) provide a global framework for advancing health, equity, and development by 2030.^1^ Health-related SDGs and the social determinants of health (SDH) are central to this agenda and are closely interconnected with progress across many other goals.^2^^,^^3^ India, home to more than 1.4 billion people, will play a pivotal role in determining global SDG attainment.^4^ However, national averages often conceal substantial inequalities, and progress at the national level does not ensure that all regions or population groups benefit equally.^5^^,^^6^ Consistent with the SDG commitment to “leave no one behind”,^7^ there is increasing recognition of the need for more geographically granular SDG monitoring. Recent efforts have expanded SDG monitoring beyond the national level to states, districts, and political constituencies.^4^^,^^8^ These analyses have generated important evidence for policy-making, informed targeting of interventions and course corrections, and revealed substantial shortfalls in progress toward many targets. Nevertheless, most assessments remain confined to administrative units that are too large to identify communities that are being left behind. Prior studies have documented substantial small-area variation in population health and SDH-related outcomes in India, indicating that district-level averages can mask important local inequalities and limit the ability of policymakers to effectively target interventions.^9^^,^^10^

Villages represent a particularly important unit for understanding and addressing these inequalities. As of the 2011 Census,^11^ nearly two-thirds of India's population resides in rural areas, and many health, nutrition, sanitation, and social development programs are implemented through village-level institutions and governance structures.^12^, ^13^, ^14^, ^15^, ^16^ Village-level evidence can therefore provide actionable information for local planning, resource allocation, and program targeting. Despite their policy relevance, village-level evidence remains largely absent from national SDG monitoring efforts. To address this limitation, we extend a previously developed semi-supervised framework to generate village-level estimates for a broader set of population health and SDH–related SDG indicators across 597,603 villages in India.^12^ Further, using 2019–2021 cross-sectional estimates, we provide a descriptive assessment of village-level distributions, inequalities, and target attainment for 35 SDG indicators and estimate the Required Rates of Improvement (RRI) needed to achieve the corresponding SDG targets.

## Methods

We used two data sources for this analysis. The first was the fifth round of the National Family Health Survey (NFHS-5), a nationally representative cross-sectional household survey conducted in India using a stratified two-stage sampling design.^17^ The 2011 Census served as the sampling frame for selection of primary sampling units (PSUs)—villages in rural areas and census enumeration blocks in urban areas. Within each PSU, households were systematically sampled following a complete household listing. NFHS-5 was conducted in two phases (2019–20 and 2020–21) and covered all 707 districts across 36 states and union territories (UTs) of India.

The second data source was the 2011 Census of India village-level demographic and amenities data.^11^ Demographic attributes were obtained from ML Infomap, which digitized village boundaries and settlement points using census atlases and high-resolution satellite imagery.^18^ Village amenities data were extracted from the District Census Handbooks published by the Office of the Registrar General and Census Commissioner of India. Village boundaries were obtained from the Bharat Map polygon shapefile, which provided village polygons for all states except eight: Arunachal Pradesh, Himachal Pradesh, Jammu & Kashmir, Ladakh, Manipur, Mizoram, Nagaland, and Sikkim. For these eight states, Thiessen polygons for each village were generated from village point locations using ArcGIS Pro. Demographic and amenities datasets were linked using unique village identifiers derived from state, district, subdistrict, and village codes, resulting in a harmonized dataset of 597,603 inhabited villages with complete information on population characteristics, infrastructure, and service availability. These census-based village features served as predictors in the village-level estimation framework.

We selected SDG indicators related to population health and SDH, following the framework adopted in prior district-level and political constituency assessments of SDG progress in India.^4^^,^^8^ Using indicator lists from the Demographic and Health Surveys (DHS) program and the National Institution for Transforming India (NITI Aayog), we identified 24 sub-SDGs, corresponding to 35 indicators available in the dataset (Table S1).^19^^,^^20^ Indicator definitions followed the Global Indicator Framework for the SDGs.^21^ The selected indicators spanned poverty (Goal 1), nutrition (Goal 2), health and well-being (Goal 3), gender equality (Goal 5), water and sanitation (Goal 6), energy (Goal 7), financial inclusion (Goal 8), peace, justice and strong institutions (Goal 16), and digital access and partnerships (Goal 17).

### Statistical analysis

We first used individual-level survey data to generate cluster-level estimates for each SDG indicator. For this step, we applied multilevel logistic regression models that account for the hierarchical survey design, with individuals nested within clusters, districts, and states.^9^ This modeling approach produces precision-weighted cluster estimates by pooling information across higher-level units, thereby stabilizing estimates for clusters with small sample sizes. Further details on the precision-weighted multilevel modeling strategy are provided in the eAppendix A. Consistent with the sampling design, the cluster-level models included both rural and urban PSUs. However, because the present analysis focuses on census-defined villages as the unit of interest, all subsequent village-level estimates were restricted to rural villages.

Cluster-level predictions were then linked to census villages and their characteristics. Because survey clusters are anonymized and spatially displaced to protect confidentiality, a one-to-one mapping between clusters and villages is not possible. This fuzzy mapping poses a fundamental labeling problem. Each cluster may correspond to multiple potential villages, and most villages do not have a uniquely matched cluster. To overcome this limitation, we applied an extension of a previously developed semi-supervised small-area estimation framework.^12^ We extended the earlier semi-supervised approach to jointly predict all 35 SDG indicators. The procedure consists of two components: (1) initialization, which relies only on villages that have a unique cluster–village link, and (2) iterative label refinement, which progressively improves the assignment of cluster-level estimates to villages.

In the initialization stage, census-based village features were used as predictors in a RandomForest model trained on the uniquely matched villages, yielding an initial set of village-level predictions for all indicators. Because cluster-level estimates were not available for every indicator in every cluster, the number of clusters contributing to model training varied across indicators (Table S1). Since most clusters are linked to multiple villages, we then implemented an iterative label-correction step. For each cluster, we identified the village whose predicted indicator vector was closest to the cluster-level estimates based on Euclidean distance, and assigned that village as its provisional label. The RandomForest model was retrained using this expanded labeled set, and the two steps were repeated until predictions stabilized (after 15 iterations). This framework improves upon earlier implementations by exploiting correlations across indicators during the label-expansion stage. To evaluate predictive performance, we compared district-level estimates aggregated from village predictions with district-level estimates aggregated from cluster-level predictions and calculated Pearson correlation coefficients across districts. Full mathematical details, including formal notation, algorithms, and diagnostic assessments, are provided in eAppendix B.

Village-level estimates were summarized using means, medians, standard deviations, ranges, and Gini coefficients. Gini coefficients were calculated across all villages for each SDG indicator to quantify inter-village inequality, with values ranging from 0 (equality) to 1 (inequality). To quantify within-district variation in village-level estimates, we calculated district-level means and within-district, between-village standard deviations for each indicator. We further assessed the relationship between district means and within-district, between-village variability using Pearson correlation coefficients. For analyses requiring aggregation to districts, village-level estimates were aggregated to harmonized district boundaries reflecting the most recent administrative geography in India.^9^ To assess village-level status relative to the 2030 SDG targets, we calculated the proportion of villages that had already achieved the corresponding target by 2021 and summarized the proportion achieving each target across state/UTs. For villages below the target level in 2021, we estimated the RRI, defined as the annual percentage-point change needed between 2021 and 2030 to reach the corresponding SDG target.

To facilitate visualization and interpretation of the village-level estimates, we developed an interactive web-based dashboard: https://india-sdg-village-dashboard.streamlit.app/. The dashboard provides interactive visualizations through maps and summary statistics panels. The dashboard serves as a decision-support and dissemination tool to translate the data into an accessible format for monitoring SDG progress at granular geographic scales. Additional details on dashboard use and development are provided in the eAppendix C and D, respectively.

Multilevel models used to generate cluster-level estimates were fitted in Stata 19.5 using the *runmlwin* command.^22^^,^^23^ Village-level prediction analyses and data visualization were conducted in R version 4.4.0.^24^

### Ethics statement

The NFHS was conducted with informed consent from participants, and the survey protocol, including all questionnaires, received ethical approval from the Institutional Review Boards of the International Institute for Population Sciences and ICF. The present study used only de-identified, publicly available NFHS data. Under Harvard Longwood Campus IRB guidance, researchers may use the IRB Decision Tool to determine whether a study requires institutional review. Based on this assessment, the present study was determined to be exempt from full institutional review.

### Role of funding source

The funder had no role in study design, data collection, data analysis, interpretation, or writing of the report.

## Results

The analysis included 35 SDG indicators, with cluster sample sizes ranging from 9102 to 30,170 and individual sample sizes ranging from 63,851 to 2,795,894 (Table S1). Variance partitioning analyses showed that cluster-level variation exceeded district-level variation for all 35 indicators (Table S2). For several indicators, including stunting, wasting and overweight, neonatal mortality, full vaccination coverage, partner violence, electricity access, and women's bank account ownership, more than half of the geographic variation occurred at the cluster level, suggesting that substantial heterogeneity exists within the districts (Table 1). Consistent with the variance partitioning results, substantial heterogeneity also remained between villages within the same district (Table S3). While some indicators, including stunting (median within-district, between-village SD: 1.41%), under-five mortality (0.15%), and neonatal mortality (0.10%), exhibited relatively limited within-district, between-village variability, considerably greater within-district, between-village variation was observed for clean fuel for cooking (12.42%), hand-washing facilities (11.93%), access to basic services (11.68%), and improved sanitation (11.53%).

**Table 1:** Variance partitioning of 35 Sustainable Development Goal (SDG) indicators across three geographical units of India.

Indicator number	Indicator name	Percentage of variation attributable to each level		
		State (%)	District (%)	Cluster (%)
**Goal 1:End poverty in all its forms everywhere**				
1.2.2	Multidimensional poverty	39.7	15.1	45.2
1.3.1	Health insurance (women)	69.6	6.7	23.7
1.3.1	Health insurance (men)	58.7	5.8	35.5
1.4.1	Access to basic services	32.7	14.8	52.5
**Goal 2:End hunger, achieve food security and improved nutrition and promote sustainable agriculture**				
2.2.1	Stunting	14.0	15.7	70.3
2.2.2	Wasting and overweight	16.2	13.7	70.1
2.2.3	Anemia (women)	57.7	11.8	30.5
2.2.3	Anemia (pregnant women)	36.8	14.7	48.5
2.2.3	Anemia (non-pregnant women)	58.0	11.7	30.3
**Goal 3:Ensure healthy lives and promotewell-beingfor all at all ages**				
3.1.2	Skilled birth attendance	52.6	10.4	36.9
3.2.1	Under-5 mortality	34.2	12.4	53.4
3.2.2	Neonatal mortality	31.7	10.4	57.9
3.7.1	Modern contraceptive use	65.8	9.7	24.6
3.7.2	Adolescent pregnancy (10–14)	45.3	14.9	39.8
3.7.2	Teenage pregnancy (15–19)	54.4	20.7	24.8
3.a.1	Tobacco use (women)	71.2	12.5	16.2
3.a.1	Tobacco use (men)	49.5	11.1	39.4
3.b.1	Full vaccination (card)	20.7	21.9	57.4
**Goal 5:Achieve gender equality and empower all women and girls**				
5.2.1	Partner violence (any)	33.0	10.9	56.1
5.2.1	Partner violence (emotional)	17.4	13.5	69.1
5.2.1	Partner violence (physical)	38.5	10.0	51.5
5.2.1	Partner violence (sexual)	21.0	11.6	67.4
5.3.1	Child marriage girl (<15)	48.1	21.9	30.0
5.3.1	Child marriage girl (<18)	53.0	20.0	26.9
5.b.1	Own mobile phone (women)	48.1	14.1	37.8
5.b.1	Own mobile phone (men)	36.0	11.9	52.1
**Goal 6:Ensure availability and sustainable management of water and sanitation for all**				
6.1.1	Improved water	26.2	19.7	54.1
6.2.1	Improved sanitation	45.0	14.4	40.6
6.2.1	Hand-washing facility	32.5	14.8	52.8
**Goal 7:Ensure access to affordable, reliable, sustainable and modern energy for all**				
7.1.1	Electricity access	27.8	12.7	59.4
7.1.2	Clean fuel for cooking	42.8	15.3	41.9
**Goal 8:Promote sustained, inclusive and sustainable economic growth, full and productive employment and decent work for all**				
8.10.2	Have bank account (women)	26.4	10.7	62.9
**Goal 16:Promote peaceful and inclusive societies, provide access to justice for all and build effective, accountable and inclusive institutions at all levels**				
16.2.3	Teenage sexual violence	32.6	10.2	57.2
16.9.1	Birth registration	48.4	12.3	39.3
**Goal 17:Strengthen the means of implementation and revitalize the Global Partnership for Sustainable Development**				
17.8.1	Internet use	35.3	14.5	50.1

Geographic variance was decomposed using four-level multilevel logistic regression models with random intercepts at the state, district, and cluster levels. Variance partition coefficients (VPCs) represent the percentage of total residual variance attributable to each level and were computed as the variance at each level divided by the sum of variances across all geographical units.

**Fig. 1:**
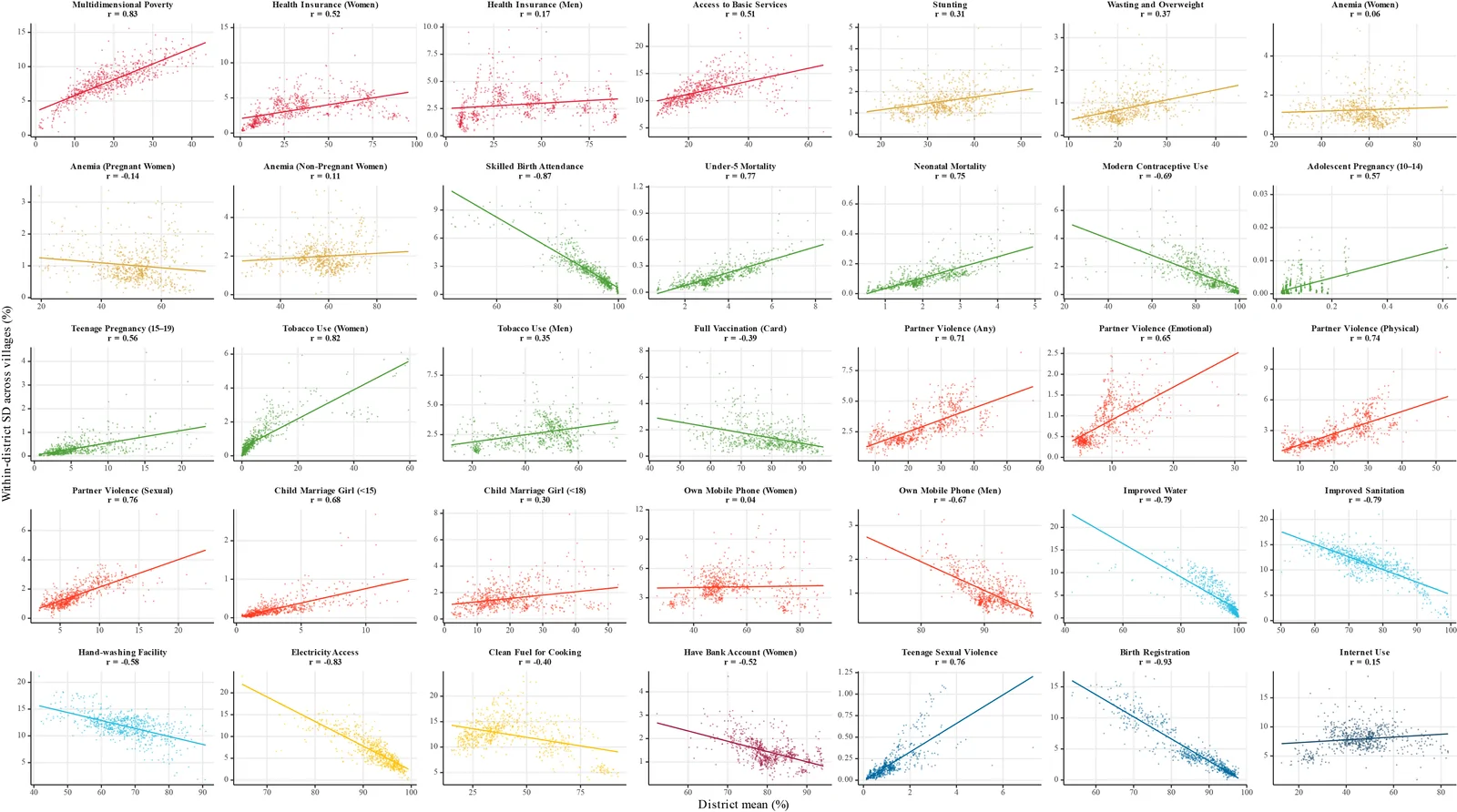
**Between-village, within-district variability by district-level mean for 35 Sustainable Development Goal (SDG) indicators in India.***Note*. Each point represents a district. The x-axis shows the district-level mean value and the y-axis shows the within-district, between-village standard deviation. Solid lines represent fitted linear regression lines. Pearson correlation coefficients are shown for each indicator. Analyses were based on 709 districts; one district containing a single village was excluded because within-district variability could not be estimated.

The relationship between district-level indicator values and within-district, between-village heterogeneity showed contrasting patterns according to indicator direction (Fig. 1). Among adverse indicators, higher district means were generally associated with greater within-district, between-village heterogeneity. The strongest positive correlations were observed for multidimensional poverty (r = 0.83), tobacco use among women (r = 0.82), under-five mortality (r = 0.77), partner sexual violence (r = 0.76), teenage sexual violence (r = 0.76), and neonatal mortality (r = 0.75). Thus, districts with a greater average burden of these outcomes also tended to exhibit greater heterogeneity between villages. Conversely, for several indicators of service coverage and access, higher district means were associated with lower within-district, between-village heterogeneity. Strong inverse correlations were observed for birth registration (r = −0.93), skilled birth attendance (r = −0.87), electricity access (r = −0.83), and improved water and improved sanitation (both r = −0.79). For these indicators, districts with lower average coverage tended to exhibit greater village-level heterogeneity. This pattern was not universal as correlations were weak for anemia among women (r = 0.06), women's mobile-phone ownership (r = 0.04), health insurance among men (r = 0.17), and internet use (r = 0.15).

**Fig. 2:**
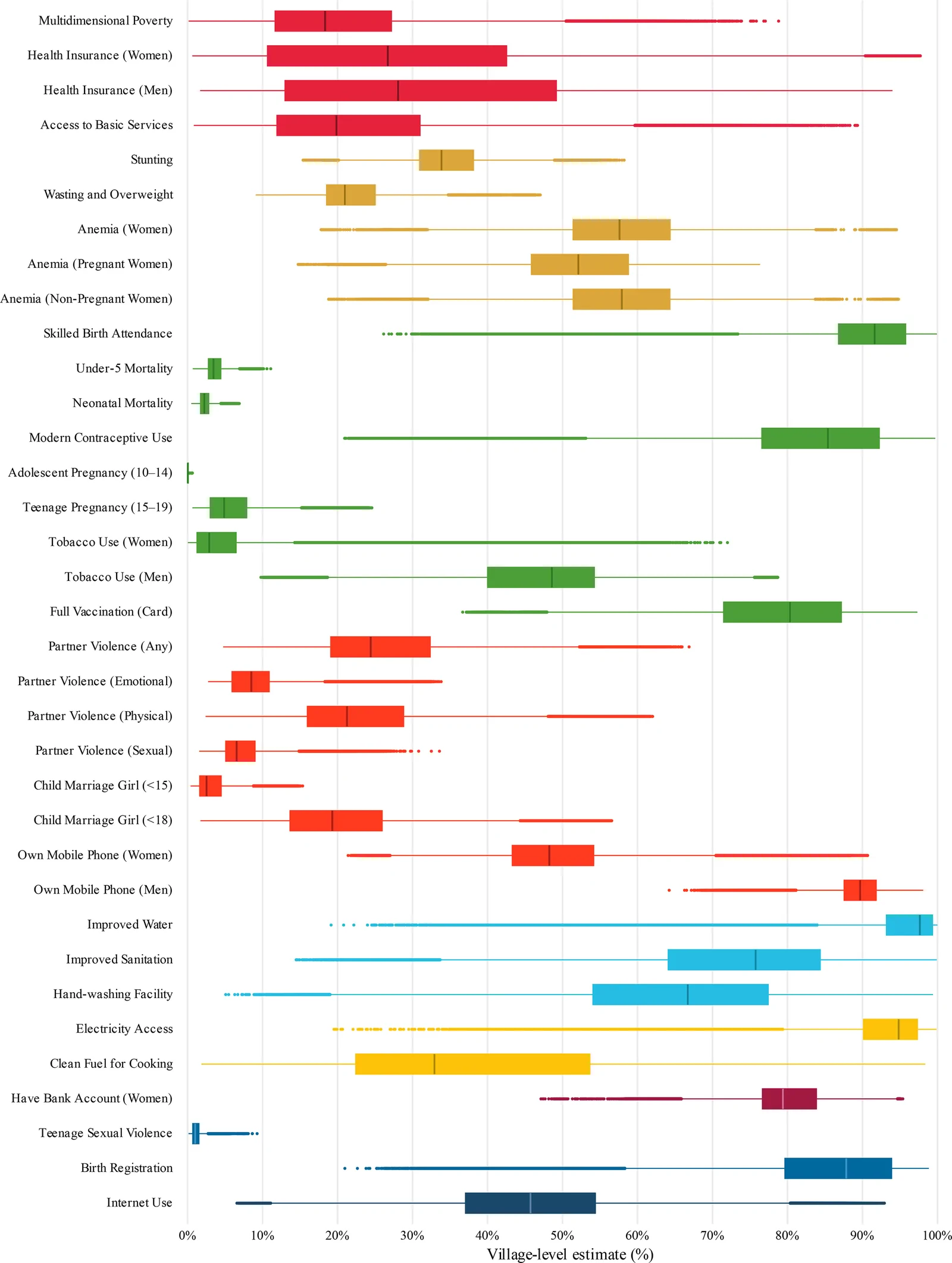
**Box-plot distribution of estimates for 35 Sustainable Development Goal (SDG) indicators across villages in India.***Note*. Boxes represent the interquartile range (IQR), center lines indicate medians, and whiskers extend to the most extreme values within 1.5 times the IQR, and points beyond the whiskers represent outliers.

Substantial variation was also observed across villages for most SDG indicators (Table 2; Fig. 2). Village-level estimates ranged from 0.14% to 78.82% for multidimensional poverty, from 1.67% to 94.00% for health insurance coverage among men, and from 6.58% to 92.93% for internet use. Several indicators exhibited highly skewed distributions, with median values below corresponding means, most notably tobacco use among women (mean 5.05%, median 2.89%), teenage pregnancy (6.21% vs 4.87%), and clean fuel for cooking (38.52% vs 32.92%), indicating that a relatively small number of villages experienced substantially higher levels than the majority. Inter-village inequality also varied considerably across indicators. Gini coefficients were highest for tobacco use among women (0.56), adolescent pregnancy among girls aged 10–14 years (0.46), health insurance coverage among women (0.41), health insurance coverage among men (0.40), and child marriage before age 15 (0.39), indicating substantial disparities across villages. In contrast, inequality was comparatively low for men's mobile phone ownership (0.02), improved water access (0.04), electricity access (0.04), skilled birth attendance (0.04), and women's bank account ownership (0.04).

**Table 2:** Distribution of village-level estimates and Gini coefficients for 35 Sustainable Development Goal (SDG) indicators in India.

Indicator number	Indicator name	Target (%)	Mean (%)	SD (%)	Median (%)	Min (%)	Max (%)	Gini
**Goal 1:End poverty in all its forms everywhere**								
1.2.2	Multidimensional poverty	13.95	20.39	11.46	18.34	0.14	78.82	0.31
1.3.1	Health insurance (women)	99	31.65	23.99	26.71	0.63	97.71	0.41
1.3.1	Health insurance (men)	99	35.06	25.41	28.08	1.67	94.00	0.40
1.4.1	Access to basic services	99	22.87	14.16	19.85	0.83	89.34	0.34
**Goal 2:End hunger, achieve food security and improved nutrition and promote sustainable agriculture**								
2.2.1	Stunting	23.05	34.22	5.71	33.86	15.41	58.22	0.09
2.2.2	Wasting and overweight	5	21.89	5.06	20.98	9.09	47.04	0.13
2.2.3	Anemia (women)	26.55	57.88	10.07	57.60	17.80	94.53	0.10
2.2.3	Anemia (pregnant women)	25.20	52.02	9.00	52.11	14.75	76.36	0.10
2.2.3	Anemia (non-pregnant women)	26.61	58.01	9.92	57.91	18.81	94.81	0.10
**Goal 3:Ensure healthy lives and promotewell-beingfor all at all ages**								
3.1.2	Skilled birth attendance	99	90.29	7.08	91.62	26.15	99.93	0.04
3.2.1	Under-5 mortality	2.5^a^	3.66	1.22	3.46	0.69	11.09	0.18
3.2.2	Neonatal mortality	1.2^a^	2.32	0.80	2.23	0.49	6.90	0.19
3.7.1	Modern contraceptive use	99	83.69	11.54	85.39	20.94	99.71	0.08
3.7.2	Adolescent pregnancy (10–14)	0.5	0.07	0.07	0.04	0.02	0.63	0.46
3.7.2	Teenage pregnancy (15–19)	0.5	6.21	4.38	4.87	0.65	24.58	0.36
3.a.1	Tobacco use (women)	5	5.05	6.50	2.89	0.04	72.00	0.56
3.a.1	Tobacco use (men)	5	45.87	11.70	48.59	9.78	78.72	0.14
3.b.1	Full vaccination (card)	99	78.71	10.41	80.35	36.69	97.33	0.07
**Goal 5:Achieve gender equality and empower all women and girls**								
5.2.1	Partner violence (any)	0.5	25.60	9.54	24.43	4.75	66.88	0.21
5.2.1	Partner violence (emotional)	0.5	9.11	3.95	8.50	2.74	33.82	0.23
5.2.1	Partner violence (physical)	0.5	22.24	9.14	21.26	2.40	62.02	0.23
5.2.1	Partner violence (sexual)	0.5	7.45	3.37	6.55	1.55	33.59	0.24
5.3.1	Child marriage girl (<15)	0.5	3.41	2.57	2.53	0.40	15.34	0.39
5.3.1	Child marriage girl (<18)	0.5	21.29	10.82	19.30	1.72	56.56	0.28
5.b.1	Own mobile phone (women)	99	49.95	11.58	48.24	21.41	90.69	0.12
5.b.1	Own mobile phone (men)	99	89.57	3.63	89.68	64.23	98.10	0.02
**Goal 6:Ensure availability and sustainable management of water and sanitation for all**								
6.1.1	Improved water	99	94.26	8.41	97.65	19.14	99.99	0.04
6.2.1	Improved sanitation	99	73.31	14.40	75.75	14.50	99.93	0.11
6.2.1	Hand-washing facility	99	65.02	15.56	66.70	5.09	99.42	0.14
**Goal 7:Ensure access to affordable, reliable, sustainable and modern energy for all**								
7.1.1	Electricity access	99	91.88	8.81	94.83	19.52	99.88	0.04
7.1.2	Clean fuel for cooking	99	38.52	20.64	32.92	1.85	98.38	0.30
**Goal 8:Promote sustained, inclusive and sustainable economic growth, full and productive employment and decent work for all**								
8.10.2	Have bank account (women)	99	80.14	5.88	79.40	47.13	95.38	0.04
**Goal 16:Promote peaceful and inclusive societies, provide access to justice for all and build effective, accountable and inclusive institutions at all levels**								
16.2.3	Teenage sexual violence	0.5	1.25	0.89	1.02	0.13	9.28	0.36
16.9.1	Birth registration	99	84.87	11.46	87.84	20.98	98.85	0.07
**Goal 17:Strengthen the means of implementation and revitalize the Global Partnership for Sustainable Development**								
17.8.1	Internet use	99	46.11	13.18	45.73	6.58	92.93	0.16

SD = standard deviation. Values represent the distribution of predicted village-level estimates across 597,603 villages in India. Gini coefficients quantify inter-village inequality, with higher values indicating greater disparities across villages.

a For Under-5 Mortality and Neonatal Mortality, values represent deaths per 100 live births. Detailed indicator definitions, SDG targets, and analytic sample sizes are provided in Table S1.

**Fig. 3:**
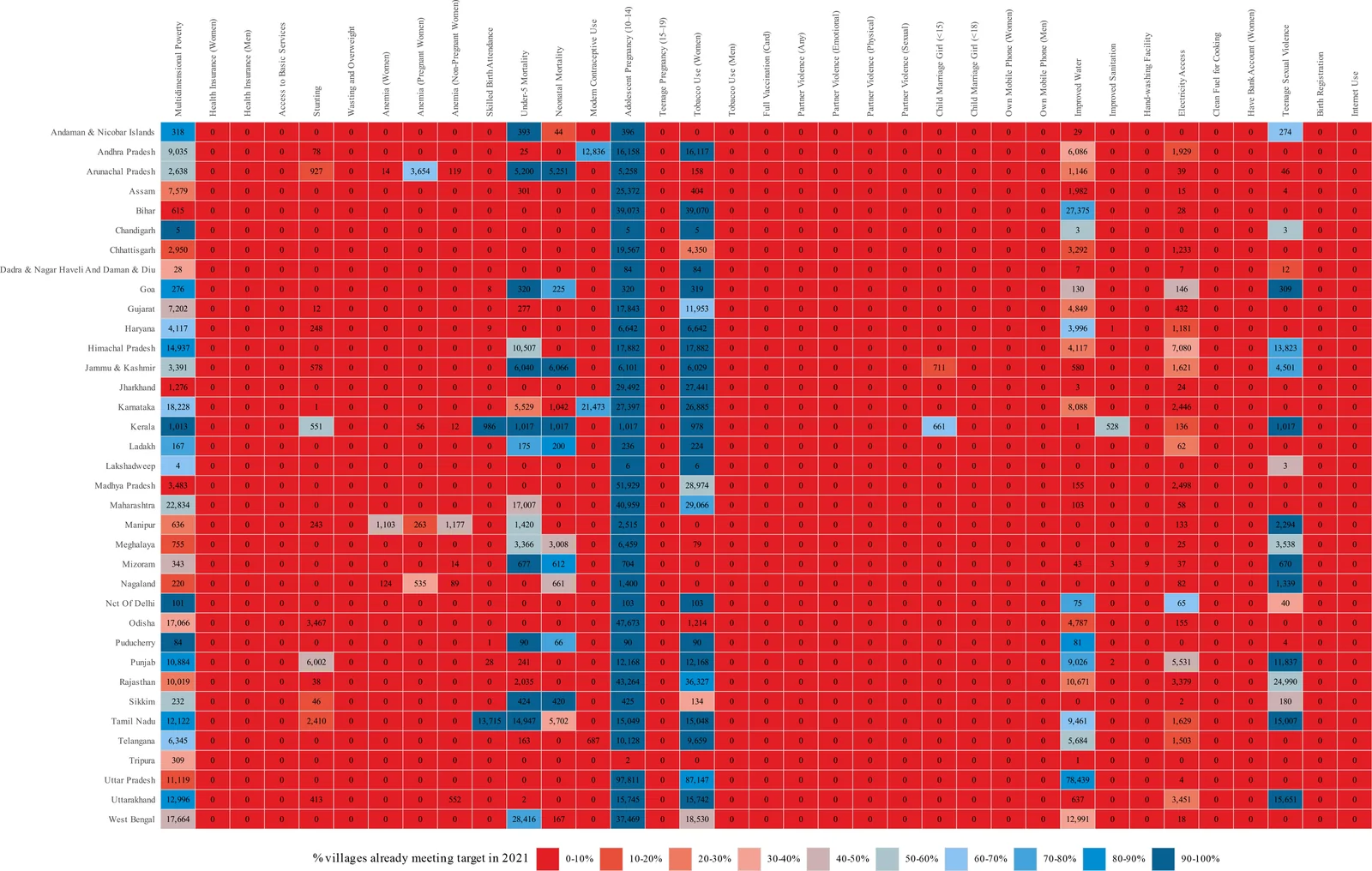
**Numberof villages already meeting the targets for 35 Sustainable Development Goal (SDG) indicators in 2021, by state and union territory in India.***Note*. Cell values indicate the number of villages within each state that had already reached the corresponding 2030 SDG target by 2021. Colors represent deciles of the percentage of villages within each state that had already reached the corresponding 2030 SDG target by 2021. Darker blue shades indicate higher percentages of villages already meeting the target, whereas darker red shades indicate lower percentages.

The proportion of villages that had already achieved the corresponding 2030 SDG targets by 2021 varied markedly across indicators (Table 3). Nearly all villages had already reached the target for adolescent pregnancy among girls aged 10–14 years (99.9%), whereas substantial proportions had also reached targets for tobacco use among women (69.1%), multidimensional poverty (33.6%), and improved water (32.4%). In contrast, no villages had yet reached targets for several indicators, including health insurance coverage, access to basic services, mobile phone ownership, women's bank account ownership, clean fuel for cooking, full vaccination coverage, internet use, and all four partner-violence indicators. Among indicators for which at least some villages had attained the target, the proportion meeting the target frequently spanned widely separated attainment bands across state/UTs, indicating that national attainment rates masked substantial geographic variation (Fig. 3).

**Table 3:** Status of 35 Sustainable Development Goal (SDG) indicators and required rates of improvement (in percentage points per year) across villages in India.

Indicator number	Indicator name	Villages already meeting the target in 2021	Required rate of improvement (% p/year)			
		N (%)	Mean	Median	Min	Max
**Goal 1:End poverty in all its forms everywhere**						
1.2.2	Multidimensional poverty	200,991 (33.6%)	1.22	0.99	0.00	6.49
1.3.1	Health insurance (women)	0 (0.0%)	6.74	7.23	0.13	9.84
1.3.1	Health insurance (men)	0 (0.0%)	6.39	7.09	0.50	9.73
1.4.1	Access to basic services	0 (0.0%)	7.61	7.92	0.97	9.82
**Goal 2:End hunger, achieve food security and improved nutrition and promote sustainable agriculture**						
2.2.1	Stunting	15,014 (2.5%)	1.15	1.10	0.00	3.52
2.2.2	Wasting and overweight	0 (0.0%)	1.69	1.60	0.41	4.20
2.2.3	Anemia (women)	1241 (0.2%)	3.14	3.11	0.00	6.80
2.2.3	Anemia (pregnant women)	4508 (0.8%)	2.71	2.70	0.00	5.12
2.2.3	Anemia (non-pregnant women)	1963 (0.3%)	3.15	3.13	0.00	6.82
**Goal 3:Ensure healthy lives and promotewell-beingfor all at all ages**						
3.1.2	Skilled birth attendance	14,747 (2.5%)	0.89	0.76	0.00	7.28
3.2.1	Under-5 mortality	98,572 (16.5%)	0.15	0.13	0.00	0.86
3.2.2	Neonatal mortality	24,481 (4.1%)	0.12	0.11	0.00	0.57
3.7.1	Modern contraceptive use	34,997 (5.9%)	1.63	1.48	0.00	7.81
3.7.2	Adolescent pregnancy (10–14)	596,742 (99.9%)	0.01	0.01	0.00	0.01
3.7.2	Teenage pregnancy (15–19)	0 (0.0%)	0.57	0.44	0.01	2.41
3.a.1	Tobacco use (women)	412,828 (69.1%)	0.69	0.44	0.00	6.70
3.a.1	Tobacco use (men)	0 (0.0%)	4.09	4.36	0.48	7.37
3.b.1	Full vaccination (card)	0 (0.0%)	2.03	1.87	0.17	6.23
**Goal 5:Achieve gender equality and empower all women and girls**						
5.2.1	Partner violence (any)	0 (0.0%)	2.51	2.39	0.42	6.64
5.2.1	Partner violence (emotional)	0 (0.0%)	0.86	0.80	0.22	3.33
5.2.1	Partner violence (physical)	0 (0.0%)	2.17	2.08	0.19	6.15
5.2.1	Partner violence (sexual)	0 (0.0%)	0.69	0.60	0.11	3.31
5.3.1	Child marriage girl (<15)	1372 (0.2%)	0.29	0.20	0.00	1.48
5.3.1	Child marriage girl (<18)	0 (0.0%)	2.08	1.88	0.12	5.61
5.b.1	Own mobile phone (women)	0 (0.0%)	4.90	5.08	0.83	7.76
5.b.1	Own mobile phone (men)	0 (0.0%)	0.94	0.93	0.09	3.48
**Goal 6:Ensure availability and sustainable management of water and sanitation for all**						
6.1.1	Improved water	193,838 (32.4%)	0.73	0.36	0.00	7.99
6.2.1	Improved sanitation	534 (0.1%)	2.57	2.33	0.00	8.45
6.2.1	Hand-washing facility	9 (0.0%)	3.40	3.23	0.00	9.39
**Goal 7:Ensure access to affordable, reliable, sustainable and modern energy for all**						
7.1.1	Electricity access	34,949 (5.8%)	0.76	0.45	0.00	7.95
7.1.2	Clean fuel for cooking	0 (0.0%)	6.05	6.61	0.06	9.72
**Goal 8:Promote sustained, inclusive and sustainable economic growth, full and productive employment and decent work for all**						
8.10.2	Have bank account (women)	0 (0.0%)	1.89	1.96	0.36	5.19
**Goal 16:Promote peaceful and inclusive societies, provide access to justice for all and build effective, accountable and inclusive institutions at all levels**						
16.2.3	Teenage sexual violence	95,542 (16.0%)	0.09	0.06	0.00	0.88
16.9.1	Birth registration	0 (0.0%)	1.41	1.12	0.01	7.80
**Goal 17:Strengthen the means of implementation and revitalize the Global Partnership for Sustainable Development**						
17.8.1	Internet use	0 (0.0%)	5.29	5.33	0.61	9.24

Villages already meeting SDG targets in 2021 (%) indicates the percentage of villages that had already reached the corresponding 2030 SDG target by 2021. Required Rates of Improvement represent the annual percentage-point change required between 2021 and 2030 to achieve the corresponding SDG target. Mean, median, minimum, and maximum values summarize the distribution of village-specific Required Rates of Improvement.

Among villages that had not yet achieved the corresponding SDG targets by 2021, the RRI varied substantially across indicators. The largest annual percentage-point improvements were required for access to basic services (7.61 percentage points per year), women's health insurance coverage (6.74), men's health insurance coverage (6.39), clean fuel for cooking (6.05), and internet use (5.29). Considerable heterogeneity was also observed within indicators. For example, the RRI for women's health insurance coverage ranged from 0.13 to 9.84 percentage points per year, while corresponding ranges were 0.06–9.72 for clean fuel for cooking, 0.61–9.24 for internet use, and 0.00–9.39 for hand-washing facilities.

Validation analyses demonstrated generally strong agreement between district-aggregated village estimates and district-aggregated cluster estimates across all 35 indicators (Table S4). District-level correlations ranged from 0.75 to 1.00 and were 0.90 or higher for the majority of indicators, including multidimensional poverty, health insurance coverage, modern contraceptive use, adolescent pregnancy, and men's mobile phone ownership.

## Discussion

This study provides a comprehensive village-level assessment of a broad range of SDG indicators across India. Three salient findings emerge. First, substantial heterogeneity exists across villages, indicating that national and district averages often conceal important local inequalities. Second, progress toward the SDGs remains limited for many indicators, with only a small proportion of villages having already achieved the corresponding 2030 targets by 2021. Third, many villages that remain below target levels would require substantial acceleration in progress to achieve the SDGs by 2030. Together, these findings highlight the importance of village-level monitoring for identifying populations at risk of being left behind and for informing more targeted efforts to accelerate SDG progress.^25^

The following data-related considerations should be taken into account when interpreting the findings and their implications. First, although district-level validation analyses generally showed strong agreement between village-aggregated and cluster-based estimates for most indicators, these analyses assess consistency after aggregation to the district level and do not constitute direct validation of individual village-level predictions. Differences in the number of clusters contributing to individual indicators may also contribute to variation in predictive performance across indicators. Village-level estimates should therefore be interpreted as model-based predictions rather than directly observed quantities. Second, uncertainty was not fully propagated through the prediction framework. The cluster-to-village assignment procedure used in this study is deterministic and therefore does not itself generate uncertainty. Rather, uncertainty arises primarily from the estimation of cluster-level indicators in the multilevel models. Generating village-level uncertainty intervals would require repeatedly sampling from the posterior distributions of cluster-level estimates and rerunning the prediction framework across many iterations to propagate uncertainty through the full modeling process. Future work should extend the framework to formally quantify and report uncertainty at the village level. Third, village-level estimates are constrained by the availability and timing of the underlying data sources. The village features used for prediction were obtained from the 2011 Census, whereas the outcome indicators were derived from NFHS-5 (2019–21). Consequently, prediction accuracy may be reduced in villages that experienced substantial demographic, socioeconomic, or infrastructural changes during the intervening period. Nevertheless, the 2011 Census remains the only nationally comprehensive source of village-level covariates covering all villages in India. Furthermore, village-level estimates were available for a single time point only. As a result, the RRIs presented in this study are necessarily based on village conditions in 2021 and should be interpreted as baseline benchmarks rather than fixed projections. Importantly, villages that had already reached the 2030 SDG targets by 2021 should not be assumed to have permanently secured those achievements, as progress may stagnate or reverse over time in response to changing economic, social, environmental, or policy conditions. Likewise, villages that had not yet reached the targets by 2021 may have experienced either faster or slower progress since then. Recently released NFHS-6 (2023–24) national fact sheet estimates suggest continued improvement in several SDG-related indicators since NFHS-5, including health insurance coverage, full vaccination coverage, internet use, and child nutritional outcomes.^26^ However, the underlying microdata are not yet available, and only aggregate national and state-level estimates have been released, so the extent to which these gains have occurred uniformly across villages remains unknown. As more detailed NFHS-6 data become available, village-level estimates and RRIs should therefore be updated and reassessed.

A central finding of this study is that district-level averages frequently conceal substantial local heterogeneity. Although districts are commonly used as the primary unit for SDG monitoring and planning, villages within the same district often differed markedly. Variance partitioning analyses showed that cluster-level variation exceeded district-level variation for all 35 indicators, while additional analyses revealed substantial heterogeneity among villages within the same district. For several indicators, substantial village-level variability remained within districts, underscoring the limitations of relying solely on district summaries for resource allocation and policy prioritization. These findings are consistent with previous evidence of substantial subnational heterogeneity in population health and SDH-related outcomes across India.^9^^,^^10^

The study also quantifies the magnitude of progress required to achieve the SDGs. Across most indicators examined, only a small fraction of villages had already achieved the 2030 targets by 2021, while many villages would require annual improvements far exceeding those historically observed at national or district levels.^4^ The substantial variation in RRI within the same indicator further highlights that average progress masks important differences in local need. These findings suggest that uniform district-wide or state-wide strategies are unlikely to be sufficient and that village-specific prioritization may be necessary to identify where immediate acceleration is required, where existing gains should be sustained, and where deeper structural interventions are needed.^27^^,^^28^

The findings also have important implications for ongoing efforts to localize SDG monitoring and implementation in India. The Ministry of Panchayati Raj and several state governments have increasingly emphasized local-level SDG monitoring through Panchayat Development Indices, SDG localization frameworks, and district- and village-level reporting systems. These initiatives recognize that aggregate state and national averages may obscure substantial local disparities in development outcomes. The village-level estimates presented in this study complement such efforts by providing a systematic assessment of the distribution and heterogeneity of population and health-related SDG indicators across more than 597,000 villages. Integrating village-level evidence into decentralized governance structures may strengthen alignment between data, planning, and implementation, enhance local accountability, and support more effective allocation of resources. The accompanying interactive dashboard was developed to support this objective by enabling policymakers and local stakeholders to visualize village-level SDG estimates and identify areas requiring targeted action. Beyond documenting geographic heterogeneity, future work should examine the socioeconomic patterning of village-level inequalities. This may help identify whether adverse outcomes are disproportionately concentrated among more marginalized villages, provide additional insight into the social distribution of progress toward the SDGs, and help guide more equitable policy responses.

At the same time, predictive approaches should not be viewed as substitutes for robust monitoring systems. The need to rely on predictive modeling at this scale reflects broader limitations in the availability of timely, high-quality local data for monitoring SDG progress. Establishing ambitious development targets without corresponding investments in data systems may undermine the ability to accurately assess progress, evaluate interventions, and guide policy action. Accordingly, the present estimates should be viewed as complementary to, rather than replacements for, strengthened local data collection, ground validation, and monitoring efforts.

In summary, the findings reveal substantial heterogeneity in SDG-related outcomes across villages and suggest that important disparities may be obscured by more aggregated forms of monitoring. By 2021, only a small proportion of villages had already achieved the 2030 SDG targets for most indicators, indicating that substantial acceleration will be required in many villages over the remainder of the SDG period. While the estimates should be interpreted in light of the study's limitations, they provide a useful baseline for understanding local variation in SDG-related outcomes and for informing future efforts to strengthen subnational monitoring and planning. Addressing these gaps will require not only more geographically granular evidence to support local planning and prioritization, but also stronger local data and monitoring systems capable of tracking progress, sustaining gains where targets have already been achieved, and identifying communities at risk of being left behind.

## Contributors

Conceptualization and Design: SVS, RK.

Data Acquisition and Analysis: SK, AB.

Data Interpretation: SK, AB, AS, JCB, SR, WJ, SVS, and RK.

Writing of the Manuscript: SK, AB, SVS, and RK.

Critical Revisions: SK, AB, AS, JCB, SR, WJ, SVS, and RK.

Supervision: SVS, RK.

SK, AB, and RK accessed and verified the underlying data reported in the manuscript. All authors had full access to all the data in the study and accept responsibility for the decision to submit for publication.

## Data sharing statement

This study is based on publicly available data from India's National Family Health Survey (NFHS-5) that are available for download through the Demographic and Health Surveys program's data distribution system: www.dhsprogram.com. The data on GPS coordinates for the Indian DHS survey clusters are available only via special request. The 2011 Census village demographics data were obtained from ML Infomap, and are available from https://www.mlinfomap.com/map-data.php. Village boundaries were obtained from the Bharat Map polygon shapefile (https://bharatmaps.gov.in/BharatMapsMVC/); for the eight states without village polygons, Thiessen polygons for each village were generated from village point locations using ArcGIS Pro. The 2011 Census village amenities data are publicly available from the Office of the Registrar General and Census Commissioner, Government of India, https://censusindia.gov.in/census.website/en. An interactive data visualization of village-level prevalence estimates, SDG target attainment status in 2021, and Required Rates of Improvement (RRI) is provided through the accompanying interactive dashboard (https://india-sdg-village-dashboard.streamlit.app/; see also eAppendix C). Derived village-level, cluster-level, and district-level estimates generated in this study are publicly available through the Harvard Dataverse repository (https://doi.org/10.7910/DVN/GO8W1W).

## Declaration of generative AI

During the preparation of this work, the authors used ChatGPT (OpenAI) to improve the readability and language of the manuscript, and OpenAI Codex to assist in developing the interactive dashboard (see eAppendix D). The authors subsequently reviewed, verified, and edited the output and take full responsibility for the content of the published article.

## Editor note

The Lancet Group takes a neutral position with respect to territorial claims in published maps and institutional affiliations.

## Declaration of interests

SK, AB, AS, JCB, SR, WJ, SVS, and RK declare no competing interests.

## Footnotes

Appendix A Supplementary data to this article can be found online at https://doi.org/10.1016/j.lansea.2026.100852.
