# Supplementary material for: Predicting the status of 35 sustainable development goal indicators in Indian villages: a semi-supervised machine learning approach for precision public policy
Source: Lancet Reg Health Southeast Asia. 2026 Sep 8;54:100852. doi: 10.1016/j.lansea.2026.100852 (PMC13578475; doi:10.1016/j.lansea.2026.100852)
Supplement: Supplementary file 1 [file mmc1.pdf]

List of Supplementary Materials

Appendix A. Precision-weighted Cluster-level Estimation Using Multilevel Models 2

Appendix B. Semi-supervised Prediction Framework 4

Appendix C. Using the Interactive Geovisualization Dashboard for 35 Sustainable Development Goal Indicators across 597,603 Villages in India, 2021 9

Appendix D. Development of the Interactive Geovisualization Dashboard for 35 Sustainable Development Goal Indicators across 597,603 Villages in India, 2021 12

Table S1. Sustainable Development Goal (SDG) indicators, definitions, targets, and analytic sample sizes 17

Table S2. Variance components (in logits), standard errors, and effective sample sizes for 35 Sustainable Development Goal (SDG) indicators across three geographical units of India 20

Table S3. District-level mean values, within-district, between-village variability, and correlations for 35 Sustainable Development Goal (SDG) indicators in India 22

Table S4. Comparison of district-aggregated cluster- and village-level estimates for 35 Sustainable Development Goal (SDG) indicators 24

Appendix A. Precision-weighted Cluster-level Estimation Using Multilevel Models

We specified a four-level logistic regression model in which individuals (level 1) are nested within survey clusters (level 2), which are nested within districts (level 3), and districts are nested within states (level 4). The model can be expressed as: $\text{logit}(\pi_{ijkl}) = \beta_0 + f_l + v_{kl} + u_{jkl}$, where π_{ijkl} denotes the probability that individual i in cluster j, district k, and state l exhibits the outcome of interest. The term β_0 represents the overall intercept, while f_l, v_{kl}, and u_{jkl} denote random intercepts at the state, district, and cluster levels, respectively. The random effects were assumed to follow normal distributions with mean zero and constant variances: $f_l \sim N(0, \sigma_f^2)$, $v_{kl} \sim N(0, \sigma_v^2)$, and $u_{jkl} \sim N(0, \sigma_u^2)$. These variance components capture geographic variation in indicator prevalence at each level of the administrative hierarchy.

Model estimation proceeded in two stages. First, we employed first-order Marginal Quasi-Likelihood (MQL1) to obtain reasonable starting values for the fixed effects and variance components. While we evaluated alternative quasi-likelihood approximations for model initialization, because higher-order Penalized Quasi-Likelihood (PQL) estimation did not consistently converge across all indicators, MQL1 was adopted as a consistent and stable approach for generating starting values. These initial estimates were then refined using Markov Chain Monte Carlo (MCMC) methods to obtain full posterior distributions for all model parameters. MCMC chains were run with burn-in periods and total iteration lengths selected to ensure adequate convergence and mixing, typically ranging from 5,000 to 25,000 iterations depending on the indicator. To improve mixing for the cluster-level variance component, we applied parameter expansion techniques. Convergence was assessed through the evaluation of effective sample sizes (ESS). For the vast majority of parameters, ESS values exceeded 250. A small number of parameters had lower ESS values. These indicators are explicitly flagged in

Table S2, which also reports ESS values for all model parameters and provides the full set of estimation results with corresponding standard errors.

Final inference was based exclusively on the MCMC posterior distributions. For each cluster-level indicator estimate, we retained the posterior mean and corresponding 95% credible interval, together with the underlying numerator and denominator used for estimation. These derived cluster-level estimates, along with the corresponding village-level predictions and district-level aggregated estimates, are made available through the Harvard Dataverse repository (https://doi.org/10.7910/DVN/GO8W1W). All multilevel models were estimated using Stata 19.5 in conjunction with MLwiN version 3.13, implemented through the *runmlwin* interface.

Appendix B. Semi-supervised Prediction Framework

In the initialization step (of Algorithm 2), the method uses villages that are mapped one-to-one with clusters as labeled data, with census features as input and the cluster-level SDG estimates as output (for all the indicators). Because cluster-level estimates were not available for every indicator in every cluster, the effective training sample size varied across indicators. A first estimate of the predictions is obtained by training a RandomForest model then computing predictions for all the villages (597,603). But since the predictions are heavily biased towards the initial labeled set (see Figure 1), we use an additional step to expand the labeled set to all the known cluster estimates.

The expand labels procedure (Algorithm 1) essentially cycles through all the clusters and maps each cluster (i.e. all it's indicators) to the village that has the smallest Euclidean distance to the village predictions (only among the villages mapped to this cluster) and hence grows the labeled set of villages to the number of clusters. This expanded set is then returned to the main procedure, and the RandomForest model is retrained on a subset of census features corresponding to this set as input and the corresponding available cluster values as output. These two steps are wrapped into an iterative scheme to further refine the expanded set and the corresponding predictions. To produce the final prediction set of 35 indicators for all 597,603 villages, we execute this two-step process 15 times or more generally when the predictions cease to change much. Intuitively our procedures can be compared to the commonly used clustering procedure referred to as K-medoids where the choice of the data point (a village in our case) representing the cluster is iteratively refined according to a cost criterion. Thus, we iteratively refine the village-cluster map to obtain a labeled set for each iteration to be used as an input to the RandomForest model. Based on a successive refinement of this procedure, we obtain a final labeled set to produce predictions. In the absence of a clean labeled set (one without a fuzzy

cluster-village mapping) the task of comparing our method to a benchmark becomes meaningless, primarily since there is no obvious available solution to problem of producing precision estimates at the village level.

Taking a closer look at the method, it was shown to be not sensitive to the choice of the initial label assignment, and we refer the reader to the univariate variant for the stunting, underweight, and wasting (SUW) indicators for a closer look. The choice of RandomForest based regression was primarily based on the well-established performance of the method, but in general we are able to use any regression-based prediction method within the framework.

In a previous work, we produced disjoint estimates for three anthropometric indicators (SUW), using a univariate procedure. The procedure developed in this work is a significant enhancement. The original SUW procedure missed out on the correlation between the indicators themselves. The current procedure predicts all the SDG metrics simultaneously by exploiting indicator correlations in the label expansion procedure where "all" the measures are considered in deciding the best closest cluster – village assignment. The original procedure involved a least squares regression-based procedure that required the output to be rescaled back to a proportion. By choosing a RandomForest based prediction model, we ensure that the output is a linear combination of existing cluster values and as such is guaranteed to be a proportion. The semi-supervised prediction framework was implemented in R version 4.4.0 using the *randomForestSRC* package.

Table 1: Notation

N	$\triangleq$	Number of Rural Villages
C	$\triangleq$	Number of Clusters
d	$\triangleq$	Number of Village Level Census Features
m	$\triangleq$	Number of Indicators to Predict
$\mathbf{V}_C$	$\triangleq$	Binary Matrix of Village Cluster Map
$\mathbf{I}_0$	$\triangleq$	Set of Villages Mapped one-to-one with Clusters
$\mathbf{Y}_C$	$\triangleq$	Matrix of Cluster Indicators
$\mathbf{X}$	$\triangleq$	Matrix of Village Census Features

Algorithm 1 Expand Labels

Input: $\mathbf{Y}_C \in \Re^{C\times m}$ ▷ Cluster Indicators
Input: $\hat{\mathbf{Y}}_\mathbf{i}$ ▷ Village Predictions
Input: $\mathbf{V}_C \in \{0,1\}^{N\times C}$ ▷ Cluster-Village Mapping
Output: $\mathbf{I}_V \in \Re^{C}$ ▷ Expanded Village Indices
  c = 0
  $\mathbf{I}_V = \Phi$ ▷ Initialize to Empty Set
  while $c < C$ **do**
    $J_c = \{j : \mathbf{V}_C[c,j] = 1\}$
    $j_{\min} = \arg\min_{j\in J_c} \left\| \hat{\mathbf{Y}}_\mathbf{i}[j,:] - \mathbf{Y}_C[c,:] \right\|$
    $\mathbf{I}_V = \mathbf{I}_V \cup j_{\min}$
  end while

Algorithm 2 Multivariate Precision Estimates

Input: $\mathbf{X} \in \Re^{N\times d}$ ▷ Village Features
Input: $\mathbf{Y}_C \in \Re^{C\times m}$ ▷ Cluster Indicators
Input: $\mathbf{V}_C \in \{0,1\}^{N\times C}$ ▷ Cluster-Village Mapping
Input: $\mathbf{I}_0$ ▷ Set of Villages Mapped one-to-one with Clusters
Output: $\hat{\mathbf{Y}}_V \in \Re^{N\times m}$ ▷ Village Indicators
  $M_0 = RandomForest(\mathbf{X}_{I_0}, \mathbf{Y}_{I_0})$ ▷ Model trained on each indicator
  $\hat{\mathbf{Y}}_0 = Predict_{M_0}(\mathbf{X}_{I_0})$ ▷ First Set of Predictions
  i = 1
  while $i \leq 15$ **do**
    $\mathbf{I}_V = ExpandLabels(\hat{\mathbf{Y}}_i, \mathbf{Y}_C, \mathbf{V}_C)$ ▷ Expanded Labels are $\Re^{C\times m}$
    $M_i = RandomForest(\mathbf{X}_{\mathbf{I}_V}, \mathbf{Y}_C)$ ▷ Refit Model on the Expanded Set
    $\hat{\mathbf{Y}}_i = Predict_{M_i}(\mathbf{X})$ ▷ Updated Predictions
    $i = i + 1$
  end while

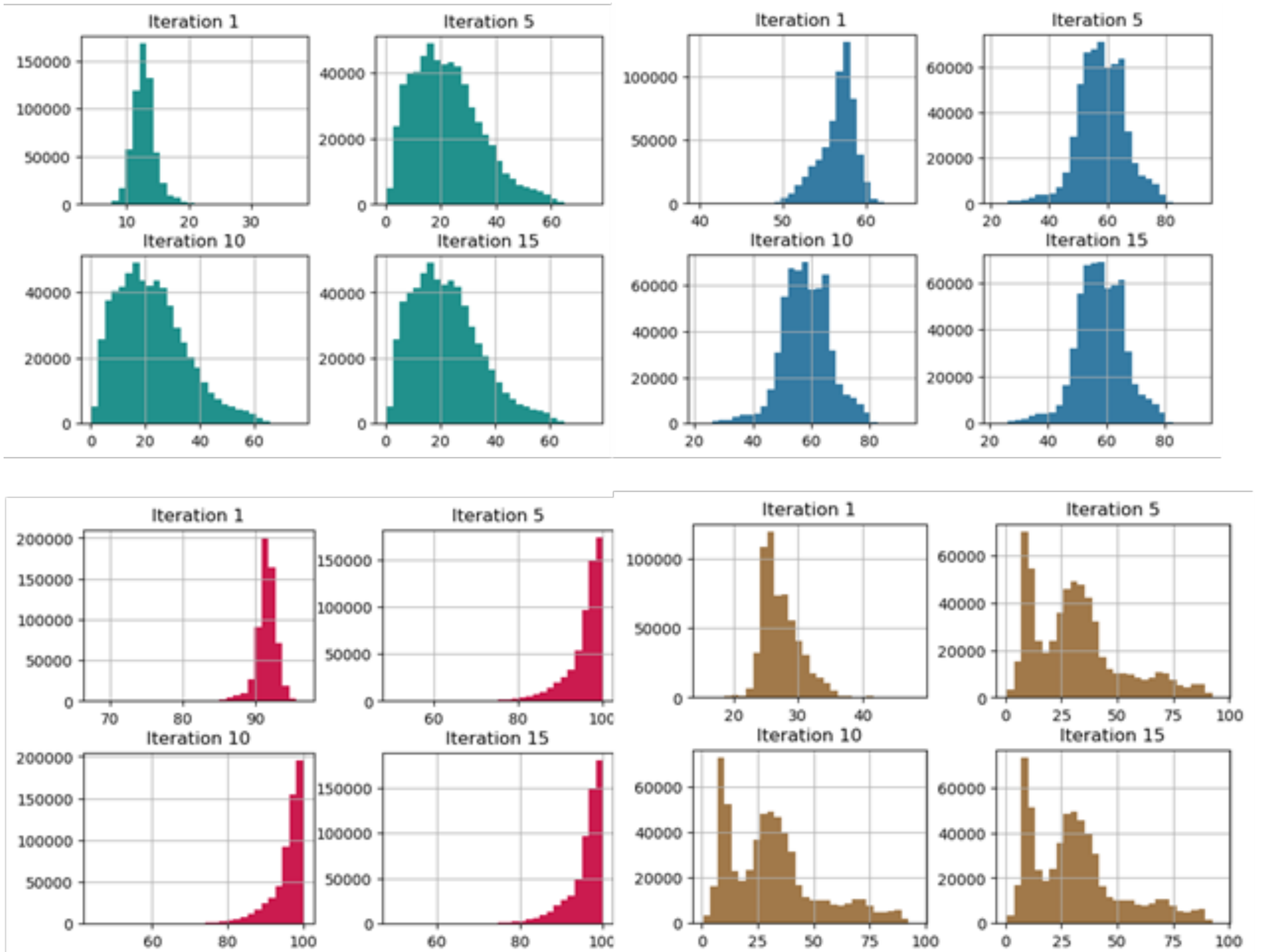

Figure 1reveals the evolution of the predictions for 4 randomly picked measures, one can see that the initial set is heavily biased towards the initial label set and as the iterations proceed the predictions converge to a final distribution.

Appendix C. Using the Interactive Geovisualization Dashboard for 35 Sustainable Development Goal Indicators across 597,603 Villages in India, 2021

This appendix outlines the key features and the steps for navigating the interactive geovisualization dashboard developed to display the village-level SDG estimates. The dashboard is available at https://india-sdg-village-dashboard.streamlit.app/.

When opened, users are presented with five selection panels on the left-hand side to choose the state, district, village, SDG indicator, and choropleth layer of interest. The center panel contains an interactive map that users can pan, zoom, and select, while the right-hand panel displays the corresponding graph and summary statistics for the selected village and indicator. Villages can also be selected directly by clicking on the desired village polygon on the interactive map. The map displays districts adjacent to the currently selected district, which can also be selected.

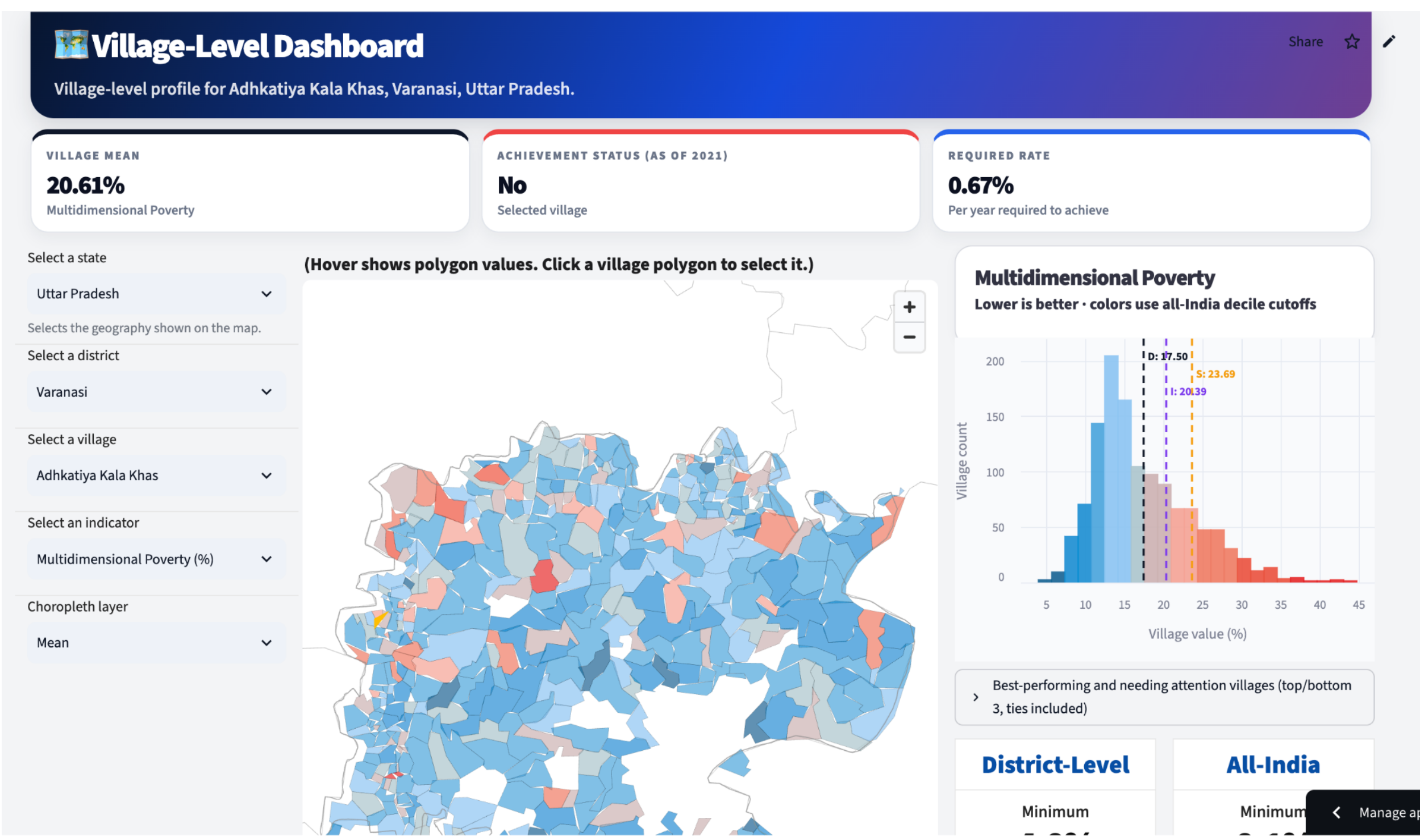

Depending on the indicator and the choropleth layer selected (Mean Value, Achievement Status as of 2021, or Required Rate of Improvement), the interactive map and statistics update accordingly. The image below showcases the view of Varanasi District when “Achievement Status as of 2021” is selected.

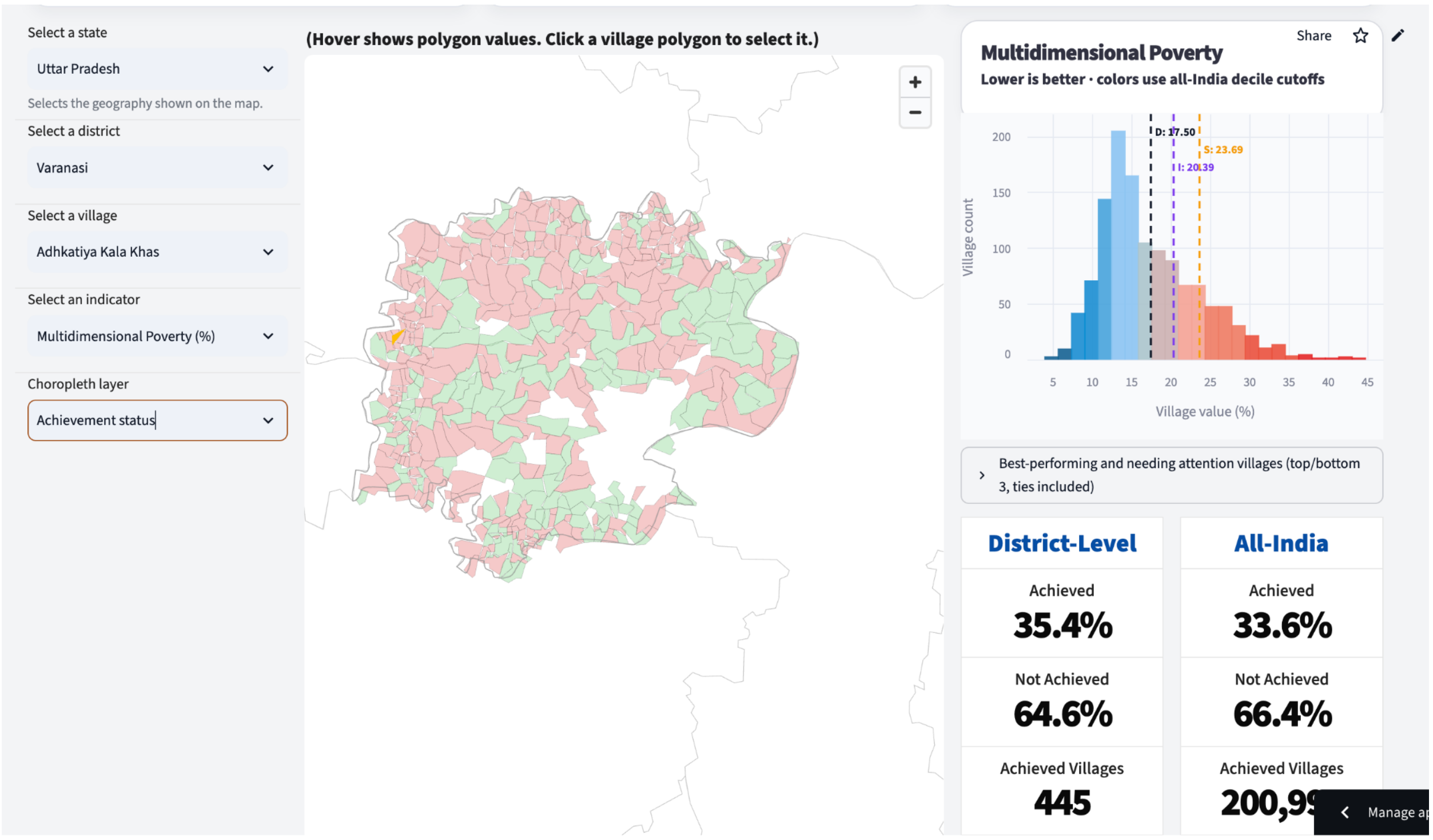

Finally, the user can also view interactive graphs for the remaining indicators for the selected village, by scrolling down to the bottom of the dashboard and toggling the button labeled "Show all indicator graphs."

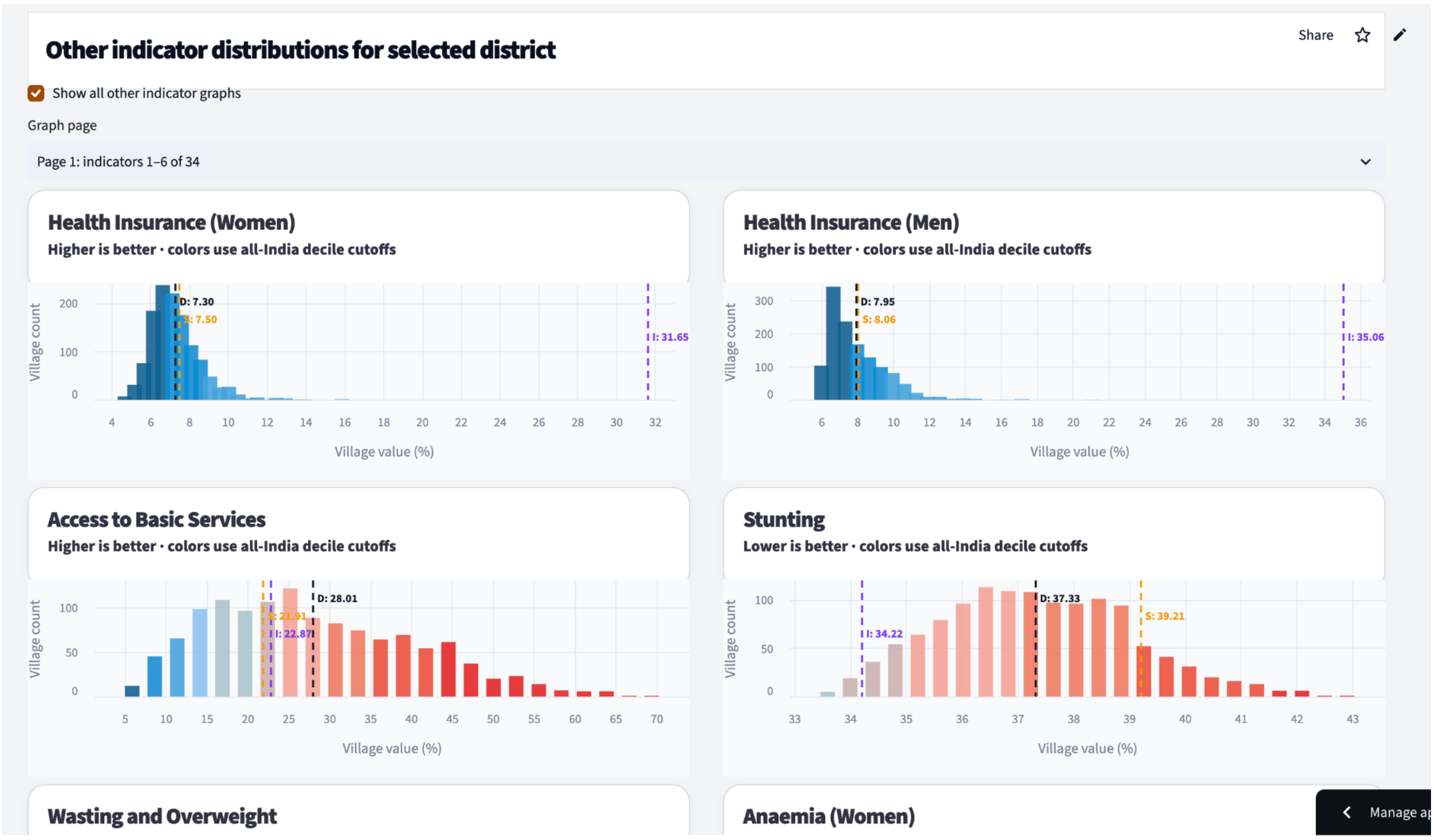

Appendix D. Development of the Interactive Geovisualization Dashboard for 35 Sustainable Development Goal Indicators across 597,603 Villages in India, 2021

Dashboard design and data specification

The interactive dashboard was built as a Streamlit web application to visualize village-, district-, and all-India-level SDG estimates. The development was done iteratively using the platform OpenAI Codex, a GPT-5 based AI coding assistant, under human supervision. Specifically, Codex was given the village-level data, district-level summary data, village and district boundary files, and screenshots of previous ArcGIS Dashboard layouts created by the researchers as design references. These screenshots were used to communicate the intended structure of the dashboard to Codex, with particular reference to the visual organization of the intended features, including geographic filtering, choropleth mapping, selected-village summaries, and district- and national-level comparisons.

The researchers then prompted Codex to analyze the input files, identify the relevant geographic identifiers and indicator fields, and propose an initial dashboard layout before generating code. This step was conducted because the dashboard required linking multiple data sources. Codex was asked to distinguish the ID and name fields at the village, district, and state levels and check their data types, uniqueness, missing or zero values, and compatibility across files, to correctly associate indicator codes with corresponding indicator names, to separate indicator mean estimates from achievement status and required rate of improvement fields, and to determine a method for joining the data records to the spatial boundary data. The village file was identified using “id_village”, “id_district722”, and “id_state”, together with their corresponding name fields. Indicator columns were identified from the prefixes “p_mean_”, “nt_”, and “rri_”; nt (achievement status) was interpreted as 1 for not achieved and 0 for achieved. The village shapefile identifier “Village__1” was converted to a numeric field

“village_id” to be matched to the data file’s field “id_village”, while District ID in the district boundary file was converted to a numeric field “district_id” for linkage to the data file’s “id_district722”. This analysis ensured that the data structure used by the application was established before the Streamlit code was written.

Application development and local testing

Following this initial step, Codex was used to generate the main Streamlit application script, app.py. The script was developed through successive researcher prompts rather than as a single automated output. The first version of app.py created the basic Streamlit interface and local preview. For local testing, a Python virtual environment was created in the project directory, with each working version launched using the command “streamlit run app.py --server.port 8502” for inspection in a web browser before deployment. Subsequent prompts then revised the application to add state, district, village, and indicator controls, render village-level choropleth maps, display results for the selected village, compute district- and all-India comparison statistics, and include distribution charts for the selected indicator. The researchers reviewed each local version and used additional prompts to correct values, labels, map behavior, and layout. The final app.py file contains the indicator dictionary, user-selection controls, map and chart construction, summary-statistic calculations, and interface styling.

Data preparation and performance optimization

Before deployment, village_prediction_final_080626.csv was read using the Python package pandas, with the village, district, and state identifiers retained as integers. The file was written using PyArrow with Zstandard compression as data/village_prediction_final_080626.parquet. The Parquet file contains the same 597,603 village records and 111 columns as the source CSV. At runtime, app.py was configured to use a column-selective Parquet read. It first reads the six

village, district, and state ID/name fields needed for the geographic selectors, and then reads only the three fields for the selected indicator (mean, achievement status, and required rate of improvement). This was done to avoid parsing all columns of the data file after every indicator change. An important step in the creation of the interactive dashboard was preparing the spatial data for optimized rendering. The village boundary layer was approximately 1.6 GB and therefore too large to load and draw in full during each dashboard interaction. To address this, the village geometries were reprojected to WGS 84 (EPSG:4326), linked to numeric village and district identifiers, simplified with topology preservation at a tolerance of 0.002 degrees, and processed into district-specific files, allowing the application to load only the villages in the selected district while retaining the complete dataset for all 597,603 villages. The output comprised 706 Zstandard-compressed GeoParquet files in data/village_geoms_by_district, each named by district ID. District boundaries were stored separately as data/district_geometries.parquet, containing 722 district features in WGS 84, so that the selected and adjacent district outlines could be drawn without loading villages outside the selected district. Codex was used to implement this district-level geometry loading, add caching, convert large inputs into deployment-ready formats, and reduce unnecessary reruns in Streamlit. These changes were necessary to reduce lag time and to increase the responsiveness of the dashboard, both in local testing and after deployment.

Validation, version control, and deployment

All major design choices, data sources, implementation, and final review were done by the researchers. Codex generated and revised code in response to researcher instructions, but the researchers determined the content of the dashboard, approved the layout, validated displayed values against the source data, and checked terminology against the manuscript. Before

deployment, python scripts/smoke_check.py was run to confirm that the required runtime files were present, that the village and district files contained the expected fields, and that sampled village and district IDs and indicator values were numeric and valid. After local testing, the relevant project files were pushed to a private GitHub repository connected to the Streamlit web application. The GitHub repository was used for version control and deployment management. Streamlit Community Cloud was configured to deploy the main branch with app.py as the application entry point. Later updates were made by editing app.py, committing the revision, and pushing it to the main branch, after which Streamlit Community Cloud automatically rebuilt and redeployed the application.

Development time and access

In total, the dashboard took ten hours to develop, including initial prototyping, data validation, debugging, performance optimization and stress testing. The dashboard is available at https://india-sdg-village-dashboard.streamlit.app/, while the code file is saved as app.py on the Harvard Dataverse repository (https://doi.org/10.7910/DVN/GO8W1W).

Village boundary data and shapefile construction

The village boundary layer was originally derived from the Bharat Map polygon shapefile. For states in which only village point locations were available, Thiessen polygons were added to construct village-level geometries as part of the spatial data processing conducted in 2023.

Village data and shapefile matching

Although the dashboard provides access to the full village-level dataset, not all villages can be displayed as polygons on the interactive map. The village-level dataset contains 597,603 unique village IDs (“id_village”). The village shapefile contains 591,721 polygon features, but only

533,062 unique non-zero village IDs. In addition, 58,659 polygons have village ID coded as 0 and therefore cannot be reliably matched to the analytic dataset using village ID.

Using “id_village” as the matching key:

- 512,265 village IDs matched between the dataset and the shapefile
- 85,338 village IDs in the dataset were not found in the shapefile
- 20,797 non-zero village IDs in the shapefile were not found in the dataset.

This incomplete linkage reflects limitations in the source boundary shapefile rather than the village-level estimates, which are complete for all 597,603 villages. Upon further analysis, the unmatched villages were not confined to a single state or district. Specifically, unmatched villages appeared across 35 of 36 states and 689 of 710 districts. We observed the largest absolute counts of unmatched villages in Uttar Pradesh, Madhya Pradesh, Assam, Rajasthan, and Maharashtra. Some districts were especially affected, including Dima Hasao in Assam and Senapati in Manipur, where all villages in the dataset were unmatched, and Karbi Anglong in Assam, where nearly all villages were unmatched.

The village-level estimates remain fully accessible through the dashboard even when a corresponding polygon is unavailable for rendering on the interactive map.

Table S1. Sustainable Development Goal (SDG) indicators, definitions, targets, and analytic sample sizes

Indicator number	Indicator name	Description	Target	Cluster sample (n)	Individual sample (n)
**Goal 1: End poverty in all its forms everywhere**					
1.2.2	Multidimensional Poverty	Proportion of men, women and children of all ages living in poverty in all its dimensions according to national definitions	13.95%	30,170	2,795,894
1.3.1	Health Insurance (Women); Health Insurance (Men)	Proportion of population covered by social protection floors/systems, by sex, distinguishing children, unemployed persons, older persons, persons with disabilities, pregnant women, newborns, work-injury victims and the poor and the vulnerable	99%	Women: 30,161; Men: 9,102	Women: 724,115; Men: 101,839
1.4.1	Access to Basic Services	Proportion of population living in households with access to basic services	99%	30,170	2,775,120
**Goal 2: End hunger, achieve food security and improved nutrition and promote sustainable agriculture**					
2.2.1	Stunting	Prevalence of stunting (height for age < –2 standard deviation from the World Health Organization Child Growth Standards median) among children under 5 years of age	40% reduction (23.05%)	29,507	206,407
2.2.2	Wasting and Overweight	Prevalence of malnutrition (weight for height > +2 or < –2 standard deviation from the World Health Organization Child Growth Standards median) among children under 5 years of age, by type (wasting and overweight)	5%	29,442	202,059
2.2.3	Anemia (Women); Anemia (Pregnant Women); Anemia (Non-Pregnant Women)	Prevalence of anemia in women aged 15–49 years, by pregnancy status (percentage)	50% reduction (All women: 26.55%; Pregnant: 25.20%; Non-pregnant: 26.61%)	All women: 30,112; Pregnant: 16,813; Non-pregnant: 30,110	All women: 690,153; Pregnant: 27,317; Non-pregnant: 662,836
**Goal 3: Ensure healthy lives and promote well-being for all at all ages**					
3.1.2	Skilled Birth Attendance	Proportion of births attended by skilled health personnel	99%	29,770	232,920
3.2.1	Under-5 Mortality	Under-5 mortality rate (per 100 live births)	2.5	29,770	232,920
3.2.2	Neonatal Mortality	Neonatal mortality rate (per 100 live births)	1.2	29,770	232,920

Indicator number	Indicator name	Description	Target	Cluster sample (n)	Individual sample (n)
3.7.1	Modern Contraceptive Use	Proportion of women aged 15–49 years whose need for family planning is satisfied with modern methods	99%	29,993	353,201
3.7.2	Adolescent Pregnancy (10–14); Teenage Pregnancy (15–19)	Adolescent birth rate (aged 10–14; aged 15–19) per 1000 women in that age group	0.5%	10–14 years: 28,655; 15–19 years: 28,655	10–14 years: 122,480; 15–19 years: 122,480
3.a.1	Tobacco Use (Women); Tobacco Use (Men)	Age-standardized prevalence of current tobacco use among persons aged 15 years and older	5%	Women: 30,161; Men: 9,102	Women: 724,115; Men: 101,839
3.b.1	Full Vaccination (Card)	Proportion of the target population covered by all vaccines included in their national program	99%	27,272	87,622
**Goal 5: Achieve gender equality and empower all women and girls**					
5.2.1	Partner Violence (Any); Partner Violence (Emotional); Partner Violence (Physical); Partner Violence (Sexual)	Proportion of ever-partnered women and girls aged 15 years and older subjected to physical, sexual or psychological violence by a current or former intimate partner in the previous 12 months, by form of violence and by age	0.5%	9,102	63,851
5.3.1	Child Marriage Girl (<15); Child Marriage Girl (<18)	Proportion of women aged 20–24 years who were married or in a union before age 15 and before age 18	0.5%	Before 15: 29,023; Before 18: 29,023	Before 15: 118,700; Before 18: 118,700
5.b.1	Own Mobile Phone (Women); Own Mobile Phone (Men)	Proportion of individuals who own a mobile telephone, by sex	99%	Women: 9,107 Men: 9,095	Women: 108,785 Men: 95,531
**Goal 6: Ensure availability and sustainable management of water and sanitation for all**					
6.1.1	Improved Water	Proportion of population using safely managed drinking water services	99%	30,170	2,795,894
6.2.1	Improved Sanitation; Hand-washing Facility	Proportion of population using (a) safely managed sanitation services and (b) a handwashing facility with soap and water	99%	Improved Sanitation: 30,170; Hand-washing Facility: 30,170	Improved Sanitation: 2,795,887; Hand-washing Facility: 2,775,127

Indicator number	Indicator name	Description	Target	Cluster sample (n)	Individual sample (n)
**Goal 7: Ensure access to affordable, reliable, sustainable and modern energy for all**					
7.1.1	Electricity Access	Proportion of population with access to electricity	99%	30,170	2,795,894
7.1.2	Clean Fuel for Cooking	Proportion of population with primary reliance on clean fuels and technology	99%	30,170	2,795,894
**Goal 8: Promote sustained, inclusive and sustainable economic growth, full and productive employment and decent work for all**					
8.10.2	Have Bank Account (Women)	Proportion of adults (15+ years) with an account at a bank or other financial institution or with a mobile-money-service provider	99%	9,107	105,968
**Goal 16: Promote peaceful and inclusive societies, provide access to justice for all and build effective, accountable and inclusive institutions at all levels**					
16.2.3	Teenage Sexual Violence	Proportion of young women and men aged 18–29 years who experienced sexual violence by age 18	0.5%	9,104	72,320
16.9.1	Birth Registration	Proportion of children under 5 years of age whose births have been registered with a civil authority, by age	99%	29,784	227,995
**Goal 17: Strengthen the means of implementation and revitalize the Global Partnership for Sustainable Development**					
17.8.1	Internet Use	Proportion of individuals using the Internet	99%	30,170	2,795,894

Note. Indicator definitions were adapted from the Global Indicator Framework for the Sustainable Development Goals and mapped to variables available in NFHS-5 (2019–21). 1.2.2 – Using Oxford's MPI Methodology and SDG Target is coming from NITI Aayog's State SDG Targets; 1.3.1 – Shows Health Insurance Coverage Only; 1.4.1 – Included Household Variables applied to all individuals and did not include Family Planning using Modern Methods; 3.7.2 – Changed to Teenage Pregnancy instead of calculating the Fertility Rate; 3.b.1 – Only included if on Vaccine Card; 6.1.1 – Applied Household Status to all Individuals within the Household; 6.2.1 – Applied Household Status to all Individuals within the Household; 7.1.1 – Applied Household Status to all Individuals within the Household; 7.1.2 – Applied Household Status to all Individuals within the Household; 16.2.3 – This only includes Women.

Table S2. Variance components (in logits), standard errors, and effective sample sizes for 35 Sustainable Development Goal (SDG) indicators across three geographical units of India

Indicator number	Indicator name	State variance (SE)	State ESS	District variance (SE)	District ESS	Cluster variance (SE)	Cluster ESS
**Goal 1:** End poverty in all its forms everywhere							
1.2.2	Multidimensional Poverty	1.89 (0.53)	461	0.72 (0.04)	542	2.16 (0.03)	651
1.3.1	Health Insurance (Women)	2.70 (0.68)	782	0.26 (0.02)	575	0.92 (0.01)	687
1.3.1	Health Insurance (Men)	2.33 (0.60)	1,073	0.23 (0.02)	302	1.41 (0.04)	624
1.4.1	Access to Basic Services	2.34 (0.65)	559	1.06 (0.07)	394	3.77 (0.04)	3,972
**Goal 2:** End hunger, achieve food security and improved nutrition and promote sustainable agriculture							
2.2.1	Stunting	0.07 (0.02)	520	0.08 (0.01)	676	0.35 (0.01)	862
2.2.2	Wasting and Overweight	0.09 (0.03)	591	0.07 (0.01)	674	0.37 (0.01)	505
2.2.3	Anemia (Women)	0.48 (0.13)	471	0.10 (0.01)	463	0.25 (0.00)	821
2.2.3	Anemia (Pregnant Women)	0.30 (0.09)	2,258	0.12 (0.01)	801	0.39 (0.05)	384
2.2.3	Anemia (Non-Pregnant Women)	0.48 (0.12)	572	0.10 (0.01)	771	0.25 (0.00)	875
**Goal 3:** Ensure healthy lives and promote well-being for all at all ages							
3.1.2	Skilled Birth Attendance	2.74 (0.76)	883	0.54 (0.04)	623	1.92 (0.04)	369
3.2.1	Under-5 Mortality	0.20 (0.06)	1,319	0.07 (0.01)	393	0.31 (0.03)	339
3.2.2	Neonatal Mortality	0.24 (0.08)	1,111	0.08 (0.01)	277	0.43 (0.04)	285
3.7.1	Modern Contraceptive Use	1.69 (0.46)	709	0.25 (0.02)	650	0.63 (0.01)	579
3.7.2	Adolescent Pregnancy (10–14)	1.24 (0.51)	11,789	0.41 (0.24)	1,303	1.09 (0.63)	216
3.7.2	Teenage Pregnancy (15–19)	0.63 (0.19)	1,842	0.24 (0.02)	1,325	0.29 (0.03)	417
3.a.1	Tobacco Use (Women)	4.33 (1.19)	707	0.76 (0.05)	463	0.99 (0.02)	439
3.a.1	Tobacco Use (Men)	0.65 (0.16)	693	0.14 (0.01)	459	0.51 (0.02)	875
3.b.1	Full Vaccination (Card)	0.31 (0.09)	581	0.33 (0.02)	640	0.85 (0.03)	335
**Goal 5:** Achieve gender equality and empower all women and girls							
5.2.1	Partner Violence (Any)	0.57 (0.17)	1,894	0.19 (0.02)	688	0.96 (0.04)	1,637
5.2.1	Partner Violence (Emotional)	0.24 (0.08)	1,999	0.19 (0.02)	673	0.97 (0.05)	1,108
5.2.1	Partner Violence (Physical)	0.77 (0.21)	2,234	0.20 (0.02)	776	1.03 (0.04)	1,359

Indicator number	Indicator name	State variance (SE)	State ESS	District variance (SE)	District ESS	Cluster variance (SE)	Cluster ESS
5.2.1	Partner Violence (Sexual)	0.41 (0.14)	2,154	0.22 (0.04)	437	1.30 (0.09)	646
5.3.1	Child Marriage Girl (<15)	0.73 (0.22)	1,705	0.33 (0.03)	898	0.45 (0.05)	338
5.3.1	Child Marriage Girl (<18)	0.71 (0.19)	1,414	0.27 (0.02)	1,555	0.36 (0.02)	546
5.b.1	Own Mobile Phone (Women)	0.68 (0.19)	582	0.20 (0.02)	457	0.53 (0.02)	861
5.b.1	Own Mobile Phone (Men)	0.35 (0.11)	746	0.12 (0.01)	280	0.51 (0.03)	413
**Goal 6:** Ensure availability and sustainable management of water and sanitation for all							
6.1.1	Improved Water	5.59 (1.57)	479	4.20 (0.40)	37	11.53 (0.22)	89
6.2.1	Improved Sanitation	3.65 (1.07)	1,061	1.17 (0.07)	566	3.30 (0.04)	1,563
6.2.1	Hand-washing Facility	1.57 (0.40)	573	0.71 (0.04)	429	2.55 (0.03)	517
**Goal 7:** Ensure access to affordable, reliable, sustainable and modern energy for all							
7.1.1	Electricity Access	2.97 (0.85)	1,721	1.36 (0.09)	550	6.34 (0.12)	275
7.1.2	Clean Fuel for Cooking	4.41 (1.21)	670	1.57 (0.09)	507	4.31 (0.04)	1,031
**Goal 8:** Promote sustained, inclusive and sustainable economic growth, full and productive employment and decent work for all							
8.10.2	Have Bank Account (Women)	0.24 (0.07)	806	0.10 (0.01)	315	0.58 (0.02)	603
**Goal 16:** Promote peaceful and inclusive societies, provide access to justice for all and build effective, accountable and inclusive institutions at all levels							
16.2.3	Teenage Sexual Violence	0.90 (0.32)	2,311	0.28 (0.07)	260	1.58 (0.17)	456
16.9.1	Birth Registration	1.65 (0.48)	931	0.42 (0.03)	495	1.34 (0.03)	431
**Goal 17:** Strengthen the means of implementation and revitalize the Global Partnership for Sustainable Development							
17.8.1	Internet Use	0.96 (0.28)	270	0.39 (0.02)	1,313	1.36 (0.01)	3,730

Note. SE = standard error; ESS = effective sample sizes. Variance components were obtained from four-level multilevel logistic regression models, with individuals nested within clusters, districts, and states.

Table S3. District-level mean values, within-district, between-village variability, and correlations for 35 Sustainable Development Goal (SDG) indicators in India

Indicator number	Indicator name	Mean district value (%)	Median within-district SD [IQR] (%)	Correlation
**Goal 1:** End poverty in all its forms everywhere				
1.2.2	Multidimensional Poverty	18.62	7.74 (6.35-9.14)	0.83
1.3.1	Health Insurance (Women)	32.71	3.27 (1.94-4.48)	0.52
1.3.1	Health Insurance (Men)	36.70	2.65 (1.82-3.64)	0.17
1.4.1	Access to Basic Services	25.61	11.68 (10.32-13.19)	0.51
**Goal 2:** End hunger, achieve food security and improved nutrition and promote sustainable agriculture				
2.2.1	Stunting	32.96	1.41 (1.12-1.80)	0.31
2.2.2	Wasting and Overweight	21.57	0.72 (0.53-1.01)	0.37
2.2.3	Anemia (Women)	56.79	1.08 (0.76-1.50)	0.06
2.2.3	Anemia (Pregnant Women)	50.54	0.89 (0.65-1.25)	-0.14
2.2.3	Anemia (Non-Pregnant Women)	56.95	1.83 (1.52-2.27)	0.11
**Goal 3:** Ensure healthy lives and promote well-being for all at all ages				
3.1.2	Skilled Birth Attendance	90.32	2.22 (1.28-3.16)	-0.87
3.2.1	Under-5 Mortality	3.40	0.15 (0.10-0.23)	0.77
3.2.2	Neonatal Mortality	2.09	0.10 (0.06-0.14)	0.75
3.7.1	Modern Contraceptive Use	84.86	1.05 (0.56-1.90)	-0.69
3.7.2	Adolescent Pregnancy (10–14)	0.07	0.00 (0.00-0.00)	0.57
3.7.2	Teenage Pregnancy (15–19)	5.78	0.24 (0.13-0.40)	0.56
3.a.1	Tobacco Use (Women)	6.11	0.68 (0.34-1.23)	0.82
3.a.1	Tobacco Use (Men)	43.42	2.43 (1.70-3.24)	0.35
3.b.1	Full Vaccination (Card)	77.91	1.16 (0.83-1.72)	-0.39
**Goal 5:** Achieve gender equality and empower all women and girls				
5.2.1	Partner Violence (Any)	24.18	2.62 (1.90-3.68)	0.71
5.2.1	Partner Violence (Emotional)	8.79	0.66 (0.40-1.18)	0.65
5.2.1	Partner Violence (Physical)	20.68	2.39 (1.78-3.50)	0.74
5.2.1	Partner Violence (Sexual)	6.97	1.38 (1.00-1.97)	0.76

Indicator number	Indicator name	Mean district value (%)	Median within-district SD [IQR] (%)	Correlation
5.3.1	Child Marriage Girl (<15)	3.06	0.17 (0.10-0.28)	0.68
5.3.1	Child Marriage Girl (<18)	19.45	1.38 (0.99-1.88)	0.30
5.b.1	Own Mobile Phone (Women)	53.30	3.99 (3.20-4.75)	0.04
5.b.1	Own Mobile Phone (Men)	90.35	0.90 (0.73-1.29)	-0.67
**Goal 6:** Ensure availability and sustainable management of water and sanitation for all				
6.1.1	Improved Water	94.03	2.50 (1.11-6.47)	-0.79
6.2.1	Improved Sanitation	75.70	11.53 (9.90-13.03)	-0.79
6.2.1	Hand-washing Facility	67.04	11.93 (10.65-13.07)	-0.58
**Goal 7:** Ensure access to affordable, reliable, sustainable and modern energy for all				
7.1.1	Electricity Access	92.30	5.75 (3.89-8.60)	-0.83
7.1.2	Clean Fuel for Cooking	42.87	12.42 (10.71-14.16)	-0.40
**Goal 8:** Promote sustained, inclusive and sustainable economic growth, full and productive employment and decent work for all				
8.10.2	Have Bank Account (Women)	80.20	1.34 (1.09-1.71)	-0.52
**Goal 16:** Promote peaceful and inclusive societies, provide access to justice for all and build effective, accountable and inclusive institutions at all levels				
16.2.3	Teenage Sexual Violence	1.08	0.13 (0.07-0.20)	0.76
16.9.1	Birth Registration	85.95	3.39 (1.67-5.57)	-0.93
**Goal 17:** Strengthen the means of implementation and revitalize the Global Partnership for Sustainable Development				
17.8.1	Internet Use	46.00	7.83 (6.78-9.00)	0.15

Note. SD = standard deviation; IQR = interquartile range. District means were calculated by aggregating village-level estimates within each district. Within-district variability was quantified using the standard deviation of village-level estimates within each district. Median within-district SD (IQR) summarizes the distribution of district-specific village-level heterogeneity. Correlations were calculated between district mean values and within-district SDs across districts. Analyses were based on 709 districts; one district containing a single village was excluded because within-district variability could not be estimated.

Table S4. Comparison of district-aggregated cluster- and village-level estimates for 35 Sustainable Development Goal (SDG) indicators

Indicator number	Indicator name	Cluster mean (%)	Village mean (%)	Correlation
**Goal 1:** End poverty in all its forms everywhere				
1.2.2	Multidimensional Poverty	17.44	18.60	0.90
1.3.1	Health Insurance (Women)	32.71	32.70	0.98
1.3.1	Health Insurance (Men)	36.67	36.69	0.98
1.4.1	Access to Basic Services	29.16	25.67	0.80
**Goal 2:** End hunger, achieve food security and improved nutrition and promote sustainable agriculture				
2.2.1	Stunting	32.73	32.95	0.94
2.2.2	Wasting and Overweight	21.43	21.55	0.97
2.2.3	Anemia (Women)	56.54	56.79	0.98
2.2.3	Anemia (Pregnant Women)	50.37	50.53	0.97
2.2.3	Anemia (Non-Pregnant Women)	56.80	56.95	0.96
**Goal 3:** Ensure healthy lives and promote well-being for all at all ages				
3.1.2	Skilled Birth Attendance	90.80	90.33	0.94
3.2.1	Under-5 Mortality	3.33	3.40	0.98
3.2.2	Neonatal Mortality	2.04	2.09	0.98
3.7.1	Modern Contraceptive Use	84.76	84.86	0.98
3.7.2	Adolescent Pregnancy (10–14)	0.07	0.07	1.00
3.7.2	Teenage Pregnancy (15–19)	5.75	5.77	0.99
3.a.1	Tobacco Use (Women)	5.93	6.11	0.99
3.a.1	Tobacco Use (Men)	42.79	43.42	0.94
3.b.1	Full Vaccination (Card)	77.94	77.91	0.97
**Goal 5:** Achieve gender equality and empower all women and girls				
5.2.1	Partner Violence (Any)	23.93	24.16	0.90
5.2.1	Partner Violence (Emotional)	8.75	8.78	0.93
5.2.1	Partner Violence (Physical)	20.48	20.67	0.90
5.2.1	Partner Violence (Sexual)	6.79	6.97	0.85

Indicator number	Indicator name	Cluster mean (%)	Village mean (%)	Correlation
5.3.1	Child Marriage Girl (<15)	3.03	3.06	0.98
5.3.1	Child Marriage Girl (<18)	19.19	19.44	0.97
5.b.1	Own Mobile Phone (Women)	54.04	53.32	0.90
5.b.1	Own Mobile Phone (Men)	90.54	90.36	0.91
**Goal 6:** Ensure availability and sustainable management of water and sanitation for all				
6.1.1	Improved Water	94.77	94.04	0.86
6.2.1	Improved Sanitation	76.58	75.73	0.79
6.2.1	Hand-washing Facility	68.80	67.08	0.81
**Goal 7:** Ensure access to affordable, reliable, sustainable and modern energy for all				
7.1.1	Electricity Access	93.45	92.31	0.75
7.1.2	Clean Fuel for Cooking	45.77	42.91	0.88
**Goal 8:** Promote sustained, inclusive and sustainable economic growth, full and productive employment and decent work for all				
8.10.2	Have Bank Account (Women)	80.28	80.20	0.92
**Goal 16:** Promote peaceful and inclusive societies, provide access to justice for all and build effective, accountable and inclusive institutions at all levels				
16.2.3	Teenage Sexual Violence	1.03	1.08	0.93
16.9.1	Birth Registration	86.95	85.96	0.91
**Goal 17:** Strengthen the means of implementation and revitalize the Global Partnership for Sustainable Development				
17.8.1	Internet Use	48.47	46.04	0.86

Note. Cluster means represent district-level estimates aggregated from the predicted cluster-level estimates, whereas village means represent district-level estimates aggregated from the predicted village-level estimates. Correlations were calculated across districts using district-level aggregated values.
